# Gender-Specific Effects of Two Treatment Strategies in a Mouse Model of Niemann-Pick Disease Type C1

**DOI:** 10.3390/ijms22052539

**Published:** 2021-03-03

**Authors:** Carsten Holzmann, Martin Witt, Arndt Rolfs, Veronica Antipova, Andreas Wree

**Affiliations:** 1 Institute of Medical Genetics, Rostock University Medical Center, D-18057 Rostock, Germany; carsten.holzmann@med.uni-rostock.de; 2 Centre of Transdisciplinary Neuroscience Rostock, D-18147 Rostock, Germany; martin.witt@med.uni-rostock.de; 3 Institute of Anatomy, Rostock University Medical Center, D-18057 Rostock, Germany; veronica.antipova@medunigraz.at; 4 Centogene AG, Rostock, Am Strande 7, 18055 Rostock, Germany; arndt.rolfs@centogene.com; 5 University of Rostock, 18055 Rostock, Germany; 6 Gottfried Schatz Research Center for Cell Signaling, Metabolism and Aging, Macroscopic and Clinical Anatomy, Medical University of Graz, A-8010 Graz, Austria

**Keywords:** NPC1, mouse, lipid storage disorder, treatment, miglustat, 2-hydroxypropyl-β-cyclodextrin, allopregnanolone, body weight, brain weight, anesthesia, behavior, accelerod test, open field test

## Abstract

In a mouse model of Niemann-Pick disease type C1 (NPC1), a combination therapy (COMBI) of miglustat (MIGLU), the neurosteroid allopregnanolone (ALLO) and the cyclic oligosaccharide 2-hydroxypropyl-β-cyclodextrin (HPßCD) has previously resulted in, among other things, significantly improved motor function. The present study was designed to compare the therapeutic effects of the COMBI therapy with that of MIGLU or HPßCD alone on body and brain weight and the behavior of *NPC1*^−/−^ mice in a larger cohort, with special reference to gender differences. A total of 117 *NPC1^−/−^* and 123 *NPC1*^+/+^ mice underwent either COMBI, MIGLU only, HPßCD only, or vehicle treatment (Sham), or received no treatment at all (None). In male and female *NPC1*^−/−^ mice, all treatments led to decreased loss of body weight and, partly, brain weight. Concerning motor coordination, as revealed by the accelerod test, male *NPC1*^−/−^ mice benefited from COMBI treatment, whereas female mice benefited from COMBI, MIGLU, and HPßCD treatment. As seen in the open field test, the reduced locomotor activity of male and female *NPC1*^−/−^ mice was not significantly ameliorated in either treatment group. Our results suggest that in *NPC1*^−/−^ mice, each drug treatment scheme had a beneficial effect on at least some of the parameters evaluated compared with Sham-treated mice. Only in COMBI-treated male and female *NPC^+/+^* mice were drug effects seen in reduced body and brain weights. Upon COMBI treatment, the increased dosage of drugs necessary for anesthesia in Sham-treated male and female *NPC1*^−/−^ mice was almost completely reduced only in the female groups.

## 1. Introduction

Niemann-Pick Type C1 (NPC1) is a rare, fatal, inherited, autosomal-recessive endolysosomal storage disease caused by mutations in the *Npc1* gene located on chromosome 18q11 [1,2]. Impaired function of the mutated NPC1 protein leads to abnormal intracellular trafficking of cholesterol and other lipids as well as various cellular components, resulting in a massive accumulation of unesterified cholesterol and gangliosides GM2 and GM3 in the late endosomes/lysosomes [3,4,5]. Besides the typical hepatosplenomegaly, the pathological accumulation of free cholesterol and glycosphingolipids in the brains of NPC1 patients leads to the damage of brain tissue with progressive neurodegeneration, causing both severe motor deficits and various psychiatric and neurological symptoms [6,7,8,9,10]. Psychiatric symptoms comprise bipolar disorder, schizophrenia-like psychosis, or major depression [11]; neurological symptoms consist of ataxia, dysarthria, dysphagia, supranuclear saccade and gaze palsy, dystonia, epileptic seizures, spasticity as well as cognitive damage, ranging in severity from specific learning disorders up to mental retardation or dementia [6,12,13,14]. The clinical presentation of NPC1 is extremely heterogeneous, with the manifestation time mainly during childhood, though juvenile and adult cases have also been reported [6,15,16,17]. Likewise, the lifespan of patients with NPC varies between a few days to over 60 years, even though a majority of cases die between 10 and 25 years of age [1,18,19].

The BALB/cNctr-Npc1^m1N^/-J Jackson NPC1 mouse strain [20,21,22,23,24,25] used here partly mimics the human disease, resulting in weight loss, lipid storage, ataxia, progressive neurodegeneration marked by cerebral atrophy, hypomyelination, degeneration of cerebellar Purkinje cells [5,20,24,26], and sensory deficits [27,28,29].

To date, there is no effective therapy available to patients with this devastating disease, although research into possible disease-modifying therapies has been ongoing since the 1950s; one main goal should be to develop treatments in order to minimize both general symptoms and neurodegeneration [30,31,32]. Because of the lack of a causal therapy so far, the imminosugar miglustat (MIGLU; N-butyldeoxynojirimycin; Zavesca^®^, Actelion Pharmaceuticals, Allschwil, Switzerland) working as a substrate reduction agent is the only authorized drug in Europa, Canada, and Japan for the treatment of the NPC1 disease, dealing with progressive neurological manifestations in both adults and children [33,34,35]. MIGLU is a small molecule able to cross the blood–brain barrier, which allows it to access malfunctioning neurons in the brain [36]. It also inhibits the synthesis of glycosphingolipids [37,38]. In the murine models of NPC, MIGLU has been shown to reduce neuronal glycosphingolipid accumulation, delay the onset of neurological dysfunction, and prolong survival of the animals [39,40]. In patients, depletion of glycosphingolipids by MIGLU reduces pathological lipid storage, improved endosomal uptake, and normalized lipid trafficking in peripheral blood B lymphocytes, improving the clinical symptoms [41,42,43]. Additionally, MIGLU is believed to reduce oxidative stress, and in the course of long-term therapy, it is well tolerated and increases lifespan and stabilizes neurologic functions in NPC1 mice [44]. Moreover, MIGLU has been shown to be an effective drug in individuals with Gaucher disease [45,46]. At the same time, MIGLU-treated patients complain of side-effects such as diarrhea, weight decrease, abdominal pain, flatulence, and tremors [47]. 

Another seemingly promising drug, the neurosteroid allopregnanolone (ALLO), is used as a replacement due to the known decreased neurosteroidogenesis in *NPC1^−/−^* mice [48]. ALLO application increased the survival of Purkinje neurons in cerebellar cultures from newborn (P0) mutant mice. Adding ALLO to the drinking water slightly increased the lifespan of *NPC1^–/–^* mutant mice from 67 to 80 d, however, locomotor function and coordination declined at eight weeks in both untreated and ALLO-treated *NPC1^−/−^* mice [48].

A subsequent study by Ahmad et al. [49] showed that ALLO treatment significantly reduces microglial activation and increases neuronal survival. Additionally, the effects on survival and weight loss of a single injection on postnatal day 7 followed by injections every two weeks were found to be more beneficial than a single injection on postnatal day 7 [49]. Interestingly, both aforementioned studies solubilized the neurosteroid, which is nearly insoluble in saline, in the sterol chelator 2-hydroxypropyl-ß-cyclodextrin (HPßCD) [48,49]. Additionally, Davidson et al. (2009) reported that administration of ALLO solubilized in HPßCD to *NPC1^−/−^* mice at postnatal day 7 was beneficial; the treated mice exhibited delayed clinical onset, extended life span, and reduced ganglioside accumulation.

Remarkably, administration of HPßCD has the same impact on ameliorating disease progression in *NPC1^−/−^* mice as the administration of ALLO solubilized HPßCD [34]. These findings are further supported by recently published works in which a single injection of ALLO in HPßCD given to mice did not increase lifespan beyond those mice receiving HPßCD alone [50,51].

Single or multiple doses of HPßCD increased the lifespan of *NPC1^−/−^* mice, improved the liver and CNS morphology and, applied alone, significantly ameliorated NPC disease [34,50,51,52,53]. According to Ramirez et al. [54], weekly administration of HPßCD overcomes the lysosomal transport defect associated with the *NPC1* mutation, nearly normalizes hepatic and whole animal cholesterol pools, prevents the development of liver disease as well as slows down cerebellar neurodegeneration, but has little or no effect on the development of progressive pulmonary disease [55,56]. HPßCD administration reverses the cholesterol transport defect seen in the *NPC1^−/−^* mice at any age, and this reversal allows the sequestered sterol to be excreted from the body as bile acid [52]. In addition, cyclic oligosaccharides are known to extract cholesterol from the plasma membrane of a variety of cells in vitro [57,58,59]. Therefore, it seems clear that HPßCD alone, not ALLO, was responsible for most and possibly for all of the effects of ALLO/HPßCD treatment [48,49,50,51,60].

As a result of the synergistic effects of the above-mentioned drugs that have been reported to exhibit benefits on clinical symptoms in NPC1 mice, Davidson et al. [34] recommended a combination of MIGLU, ALLO, and HPßCD. This COMBI treatment has been shown to reduce cerebellar neurodegeneration and intracellular lipid storage, resulting in the prevention of further Purkinje cell loss as well as sensory improvement and an increased lifespan of *NPC1* mutant mice [34,44,61,62,63,64,65]. Behaviorally, COMBI therapy positively influenced *NPC1^−/−^* mice with respect to motor function in open field, elevated plus maze, and accelerod tests [62]. Overviews of the several studies investigating body weight and the few studies investigating the brain weight of *NPC1^+/+^* and *NPC1^−/−^* mice with various treatments are given in Supplementary Materials Table S1, and of the respective behavioral studies in Supplementary Materials Table S2.

We had previously observed that *NPC1^−/−^* mice needed more drugs to induce anesthesia compared with wild types. In particular, female *NPC1^−/−^* mice needed more than males. Therefore, we systematically investigated the dosage of drugs needed to induce anesthesia in larger bi-gender cohorts. Generally, differences in gender-dependent characteristics of *NPC1^−/−^* mice have been rarely addressed as yet [34,65,66], seemingly because studying the outcome of therapeutic interventions needs larger cohorts of mice.

Therefore, to further unravel the role of single components in the therapeutic benefit of the COMBI medication, we investigated a total of 117 *NPC1^−/−^* (62 female, 55 male) and 122 *NPC1^+/+^* (66 female, 56 male) mice in 24 different groups (see Materials and Methods).

In all groups, body weight was evaluated, as body weight is a parameter most often studied in respective recent publications (Supplementary Materials Table S1). Brain weight was measured as in most of the 24 groups, no data are actually evaluable (Supplementary Materials Table S2). The same holds true for the brain to body weight ratio (%). As the sensitivity to anesthesia and the need for caution in anesthetic management has been argued in NPC patients, here, we studied the anesthetic consumption (mL) for inducing deep anesthesia, abolishing the between toes reflex measured, and the thereof derived parameters anesthetic/body weight (mL/g) and anesthetic/brain weight (mL/g). Additionally, the outcome of the accelerod (rpm) and open field performances (total distance, cm; relative center distance, %) have not yet been studied in the 24 groups, especially concerning gender differences.

## 2. Results

In Table 1, the measures found in the male and female *NPC1*^+/+^ and *NPC1^−/−^* mice of the untreated None groups are summarized.

### 2.1. Body Weight

**Figure 1:**
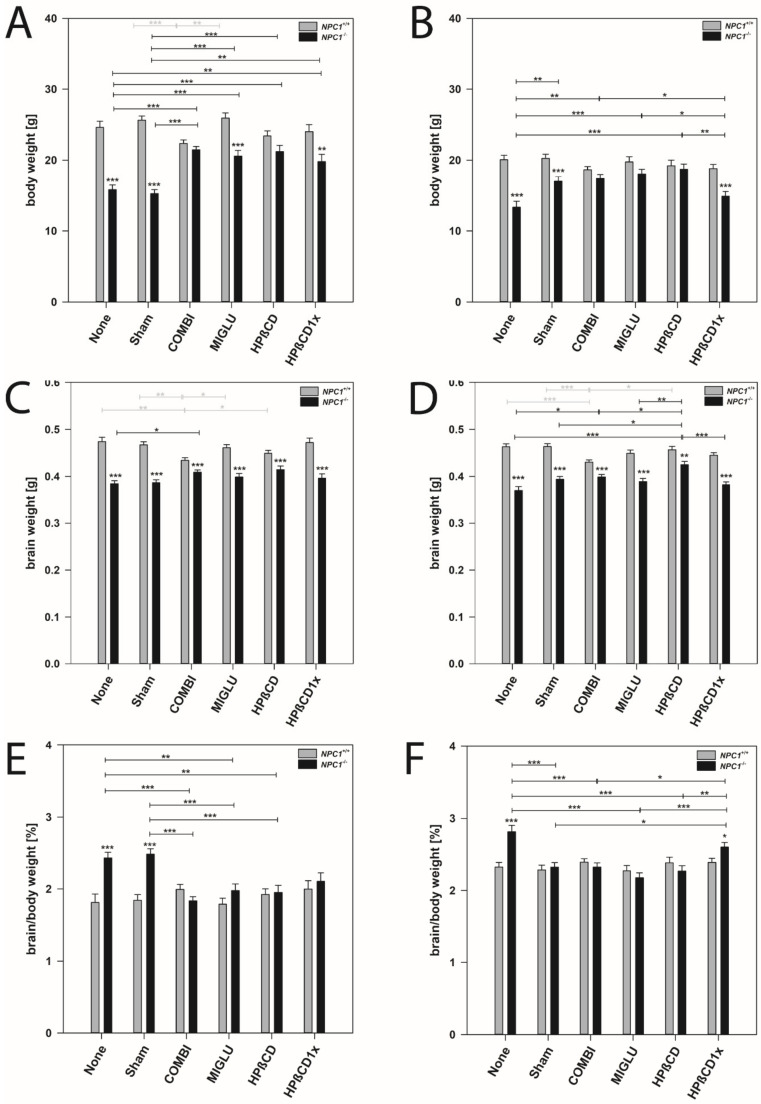
Body weight (**A**,**B**), brain weight (**C**,**D**), and quotients of brain/body weight (**E**,**F**) of *NPC1* mice. Males (**A**,**C**,**E**), females (**B**,**D**,**F**) *NPC1* mice. Significant post-hoc tests are indicated by asterisks (* *p* < 0.05, ** *p* < 0.01, *** *p* < 0.001). Data are means ± SEM.

Male mice: Wild type mice of the COMBI group had significantly reduced body weights compared to the control groups (*p* < 0.001) (Table 1 and Figure 1A). However, in all other treatment groups of wild type mice, body weight did not differ from the None or Sham groups (Figure 1A). In NPC1^−/−^ mice in all treatment groups, body weight significantly increased compared to the None or Sham groups (*p* < 0.01) (Figure 1A). Comparison of wild type and mutant mice revealed that, with the exception of the COMBI and HPßCD groups, NPC1^−/−^ mice in all treatment groups still had lower body weights than the respective wild type mice (Figure 1A).

Female mice: Wild type mice of all groups irrespective of the treatment scheme had nearly identical body weights of about 19.46 g (Figure 1B). In NPC1^−/−^ mice, except in the HPßCD1x group, significantly increased body weights were found in all treatments compared with the None group (*p* < 0.05). In the None, Sham, and HPßCD1x groups, body weights of *NPC1^−/−^* mice showed significantly lower values than the respective wild types (*p* ≤ 0.001) (Figure 1B).

### 2.2. Brain Weight

Male mice: Wild type mice of the COMBI group had significantly reduced brain weights compared to the None or Sham groups (*p* < 0.01), whereas brain weights of the MIGLU, HPßCD, and HPßCD1x groups were unchanged (Figure 1C). In *NPC1^−/−^* mice, COMBI treatment significantly increased brain weights compared to the None group (*p* < 0.05) (Figure 1C). Comparison of the brain weights of male wild type and mutant mice revealed that, irrespective of treatment, *NPC1^−/−^* mice still had relative constant brain weights (mean 0.398 g) and lower brain weights than the respective wild type mice (mean 0.460 g) (*p* < 0.001) (Figure 1C).

Female mice: Results similar to male mice were found in female mice (Figure 1D).

### 2.3. Brain Weight/Body Weight Ratio

Body weights of male mice exceeded those of females. Brain weights, however, of both sexes were in a similar range (Figure 1A–D). As body and brain weights were differently affected by the various treatment strategies, brain weight/body weight ratios were calculated.

Male mice: In wild type mice, ratios were nearly identical in all six groups investigated (mean 1.89%) (Figure 1E). In *NPC1^−/−^* mice, ratios in the None and Sham groups at about 2.46% significantly exceeded those of COMBI, MIGLU, HPßCD, and HPßCD1x groups (*p* < 0.05), altogether having lower, but nearly identical ratios in the range of 1.97% (Figure 1E).

Female mice: Ratios in all six wild type groups were nearly identical (mean 2.34%) (Figure 1F). In female *NPC1^−/−^* mice, the ratio in the None group was significantly higher (2.86%) than in the Sham, COMBI, MIGLU, and HPßCD and HPßCD1x groups (*p* < 0.05), their values being relatively constant (2.34%) (Figure 1F).

### 2.4. Anesthetic Consumption

**Figure 2:**
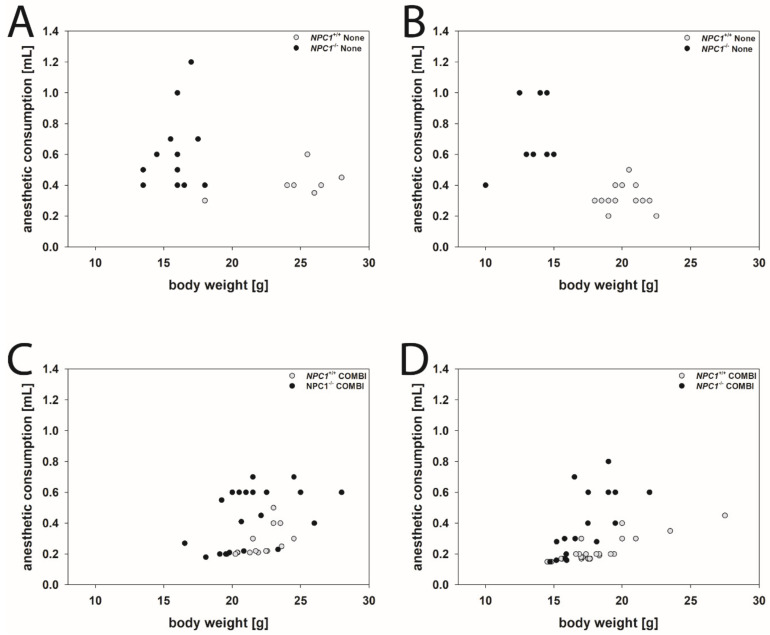
Scatter plots of anesthetic consumption compared to the body weight of None-treated males (**A**) and females (**B**) and COMBI-treated male (**C**) and female (**D**) *NPC1* mice. Open dots indicate *NPC1*^+/+^ mice, filled dots indicate *NPC1*^−/−^ mice.

As an example, for drug-induced changes of the consumption of anesthetics in *NPC1*^+/+^ and *NPC1^−/−^* mice of both genders, we needed to remove the between toes reflex. Figure 2 visualizes the effect of a COMBI treatment.

Male mice: *NPC1^−/−^* mice of the None group needed as much, or often more, drug mixture than the respective wild type group, although their body weights were considerably lower (Figure 2A). Following COMBI treatment, the body weight of *NPC1^−/−^* mice increased, however, the mean dose of the anesthetic mixture to induce anesthesia decreased. As in COMBI-treated *NPC1*^+/+^ mice, drug consumption was not significantly changed and body weight only slightly reduced, the data groups of COMBI-treated *NPC1*^+/+^ and *NPC1^−/−^* mice overlapped considerably (Figure 2C).

Female mice: Similar results were found in female mice. *NPC1^−/−^* mice of the None group required more drug mixture to induce anesthesia than the respective wild type group despite their body weights being lower (Table 1 and Figure 2B). Following COMBI treatment, the body weight of *NPC1^−/−^* mice increased; however, the mean dose of anesthetic mixture to induce anesthesia decreased. As in COMBI-treated *NPC1*^+/+^ mice, both drug consumption and body weight were not significantly changed; the data of COMBI-treated *NPC1*^+/+^ and *NPC1^−/−^* mice also overlapped (Figure 2D).

**Figure 3:**
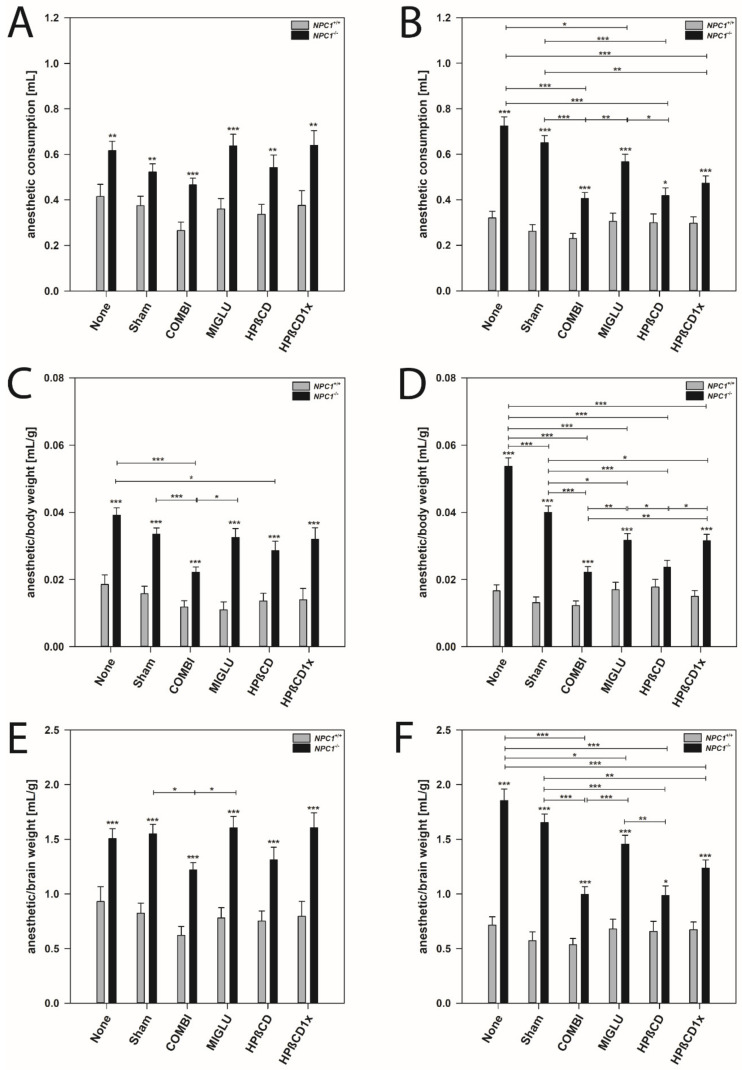
Anesthetic consumption (**A**,**B**), anesthetic consumption/body weight (**C**,**D**) and anesthetic consumption/brain weight (**E**,**F**) of *NPC1* mice. Male (**A**,**C**,**E**) and female (**B**,**D**,**F**) *NPC1* mice. Significant post-hoc tests are indicated by asterisks (* *p* < 0.05, ** *p* < 0.01, *** *p* < 0.001). Data are means ± SEM.

Additionally, other drugs induced changes of the consumption of anesthetics in *NPC1* mice, especially in females (Figure 3).

Male mice: In all wild type groups, anesthesia was induced by a similar amount of anesthetic mixture (mean 0.354 mL). Groups did not differ significantly in this parameter (*p* > 0.05) (Figure 3A). *NPC1^−/−^* mice of the None and Sham groups (mean 0.571 mL) needed a similar dosage of anesthetic mixture as all four groups injected with drugs (mean 0.572 mL) (Figure 3A). All six groups of male *NPC1^−/−^* mice needed significantly increased quanta of anesthetic mixture (mean 0.571 mL) without obvious intergroup differences compared to the wild type groups (Figure 3A).

Female mice: Anesthesia was induced in all groups of wild type mice by a comparable amount of anesthetic mixture (mean 0.286 mL). No significant intergroup differences were found (*p* > 0.05) (Figure 3B). Untreated *NPC1^−/−^* mice (None group, mean 0.725 mL) and the respective Sham group (mean 0.651 mL) needed significantly more drug mixture to abolish the between-toes reflex than all groups injected with drugs (mean 0.473 mL) (Figure 3B). In the drug-treated *NPC1^−/−^* mice, the lowest amount of drug mixture was needed by the COMBI (mean 0.406) and HPßCD groups (mean 0.418) (Figure 3B). A comparison of female wild type and mutant mice revealed significantly increased amounts of drug mixture in all *NPC1^−/−^* mice groups investigated (Figure 3B).

### 2.5. Anesthetic Consumption Related to Body Weight

Male mice: In all groups of *NPC1^+/+^* mice, the ratio of the amount of anesthetic mixture and body weight was relatively constant (mean 0.0142 mL/g) (Figure 3C). Compared to the None and Sham groups of the *NPC1^−/−^* mice (means 0.0392 mL/g and 0.0335 mL/g), the anesthetic consumption of the MIGLU- and HPßCD1x-treated groups did not change (0.0325 mL/g and 0.0320 mL/g); however, a lower dosage of anesthetic mixture was needed after both COMBI and HPßCD treatment (means 0.0222 mL/g and 0.0286 mL/g) (Figure 3C). All treatment groups of male *NPC1^−/−^* mice needed a significantly higher dosage of drug mixture per body weight (mean 0.0313 mL/g) compared to the respective wild type groups (*p* < 0.001) (Figure 3C).

Female mice: In female wild type mice, anesthesia was induced in all groups by similar amounts of anesthetic mixture (mean 0.0153 mL/g). There were no significant intergroup differences (*p* > 0.05) (Figure 3D). The various groups of *NPC1^−/−^* mice showed a quite differentiated spectrum regarding the amount of mixture needed for anesthesia (Figure 3D). Compared with the female None and Sham groups of *NPC1^−/−^* mice (means 0.0537 mL/g and 0.0400 mL/g) in all drug-treated groups, significantly decreased ratios of anesthetic mixture needed per body weight were found (mean 0.0273 mL/g) (Figure 3D). However, among the drug-treated mice, the lowest ratios were seen in the COMBI and HPßCD groups (means 0.0222 mL/g and 0.0236 mL/g) (Figure 3D). Comparison of wild type and mutant mice revealed significantly increased amounts of anesthetic mixture in all *NPC1^−/−^* mice groups investigated (*p* < 0.001), except the HPßCD group (Figure 3D).

### 2.6. Anesthetic Consumption Related to Brain Weight

Male mice: The ratio of the amount of anesthetic mixture and brain weight did not differ significantly between all groups of *NPC1^+/+^* mice (mean 0.783 mL/g) (Figure 3E). Sham and None groups of *NPC1^−/−^* mice (mean 1.528 mL/g) had ratios equal to the MIGLU, HPßCD, and HPßCD1x groups (mean 1.508 mL/g), and a significantly lower ratio was only found in the COMBI group (1.219 mL/g) (Figure 3E). Generally, male *NPC1^−/−^* mice of all six groups investigated needed significantly increased amounts of drug mixture per brain weight (mean 1.466 mL/g) compared with the respective wild type groups (mean 0.783 mL/g, *p* < 0.001) (Figure 3E). 

Female mice: In these mice, we found intergroup differences quite similar to those found for the anesthetic consumption (Figure 3F). In wild type mice, anesthesia was induced in all groups by a relatively comparable amount of drug mixture (mean 0.638 mL/g). There were no significant intergroup differences (*p* > 0.05) (Figure 3F). Compared to the female None and Sham groups of *NPC1^−/−^* mice (means 1.855 mL/g and 1.654 mL/g), all drug-treated groups showed significantly decreased ratios of drug mixture needed for anesthesia (*p* < 0.05) (Figure 3F). However, in the drug-treated mice, the lowest ratios were seen in the COMBI and HPßCD groups (means 0.996 mL/g and 0.985 mL/g) (Figure 3F). All mutant mice of all groups (mean 1.364 mL/g) showed significantly increased amounts of drug mixture per brain weight compared with wild type mice (*p* < 0.05) (Figure 3F).

**Figure 4:**
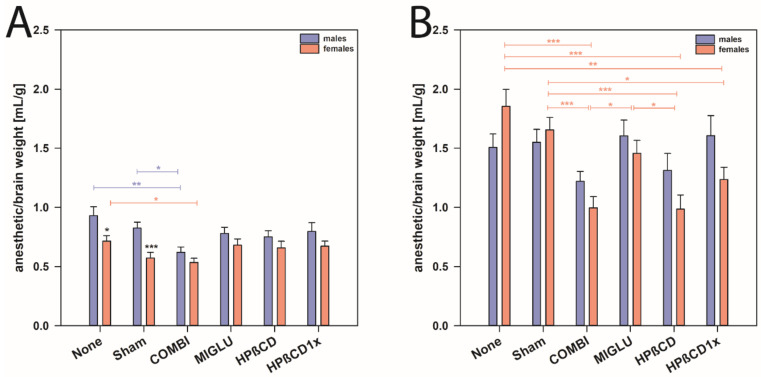
Anesthetic consumption/brain weight of male and female *NPC1* mice. (**A**) *NPC1^+/+^* and (**B**) *NPC1*^−/−^ mice. Significant post-hoc tests are indicated by asterisks (* *p* < 0.05, ** *p* < 0.01, *** *p* < 0.001). Data are means ± SEM.

Furthermore, with respect to the ratio of anesthetic solution to brain weight, we found that *NPC1^+/+^* mice of both genders needed comparable amounts of anesthetic solution (mean of all males 0.783 mL/g and mean of all females 0.638 mL/g, Figure 4A). However, female *NPC1^+/+^* mice of the None and Sham groups needed less anesthetic solution compared to the respective wild type males (mean of males from None and Sham groups 0.877 mL/g, and mean of females None and Sham groups 0.643 mL/g, *p* < 0.05, Figure 4A). 

In male *NPC1^−/−^* mice, neither treatment changed the anesthetic consumption compared to the respective None and Sham groups (Figure 4B). In female *NPC1^−/−^*, however, mice of the COMBI, HPßCD, and HPßCD1x groups needed less anesthetic solution compared to the None or Sham groups (*p* < 0.05). Obviously, various treatment options reduced the anesthetic consumption in females, but not in male *NPC1^−/−^* mice (Figure 4B).

### 2.7. Accelerod Test

**Figure 5:**
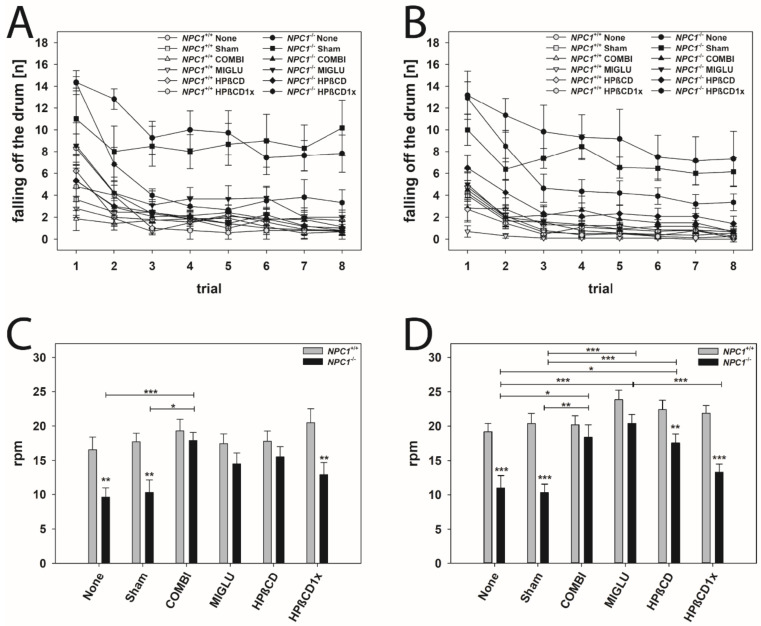
Accelerod training (**A**,**B**) and test (**C**,**D**) revealed an obvious motoric phenotype of *NPC1*^−/−^ mice that was improved by all treatment options. Male (**A**,**C**) and female (**B**,**D**) *NPC1* mice. Significant post-hoc tests are indicated by asterisks (* *p* < 0.05, ** *p* < 0.01, *** *p* < 0.001). Data are means ± SEM.

For evaluating motor coordination and balance, the accelerod test was performed (Figure 5). Mice were trained at a constant speed until they learned the task (Figure 5A,B). Animals of all groups learned the task during training trials 1 to 8, indicated by the decreasing number of fall offs in the course of the training and by reaching a constant level in the last trials (Figure 5A,B). NPC1^+/+^ male and female mice of all groups learned quickly, starting from a low level of fall offs (2 to 5 mean fall offs in 2 min), and reaching 1 to 2 fall offs in 2 min after the third training session (Figure 5A,B). Generally, NPC1^−/−^ male and female mice performed the task worse than wild types. None, Sham, and HPßCD1x groups of both male and female NPC1^−/−^ mice from the beginning of the training started at the poorest level (about 14 fall offs in 2 min) (Figure 5A,B). Moreover, these NPC1^−/−^ mice not only started at a poor level, but only reached a level of 4 to 8 fall offs in 2 min after the fourth training session (Figure 5A,B).

All groups of male and female *NPC1^+/+^* mice reached about 20 rounds per min before falling off the wheel (male: mean 18.21 rpm, female mean 21.32 rpm) (Figure 5C,D). In neither gender were significant intergroup differences found (Figure 5C,D). In mutant male mice, only the COMBI group performed significantly better than the respective None and Sham groups (Figure 5C). In female *NPC1^−/−^* mice, with the exception of the HPßCD1x group, all other treated groups (COMBI, MIGLU, and HPßCD groups) performed significantly better than those of the respective None or Sham groups (Figure 5D).

*NPC1^−/−^* male and female mice of the COMBI and MIGLU groups and also those of the male HPßCD group reached an accelerod performance comparable to the respective drug-treated wild type mice (Figure 5C,D).

**Figure 6:**
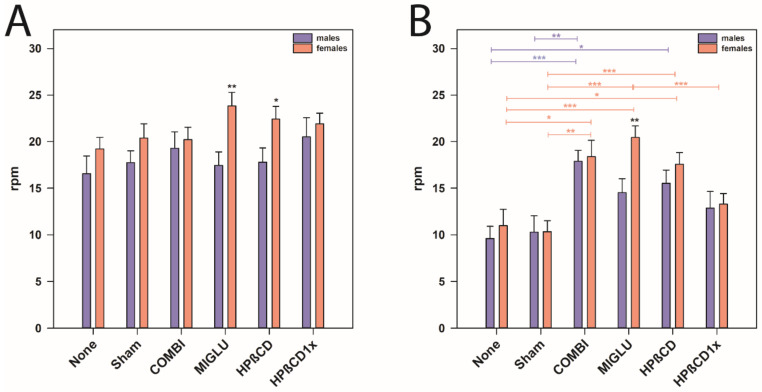
Accelerod test (**A**,**B**) revealed an obvious motoric phenotype of *NPC1*^−/−^ mice that was improved by all treatment options. However, we detected gender-specific differences of MIGLU treatment between the genotype groups. (**A**) *NPC1^+/+^* mice, (**B**) *NPC1*^−/−^ mice. Significant post hoc tests are indicated by asterisks (* *p* < 0.05, ** *p* < 0.01, *** *p* < 0.001). Data are means ± SEM.

Comparison of the accelerod of both genders revealed that female *NPC1^+/+^* mice of the MIGLU and the HPßCD groups performed better than the respective males (*p* < 0.05, Figure 6A). In *NPC1^−/−^* mice, gender-specific differences were only seen in MIGLU-treated animals, where females performed significantly better than the respective males (*p* < 0.01, Figure 6B).

### 2.8. Open Field Test

**Figure 7:**
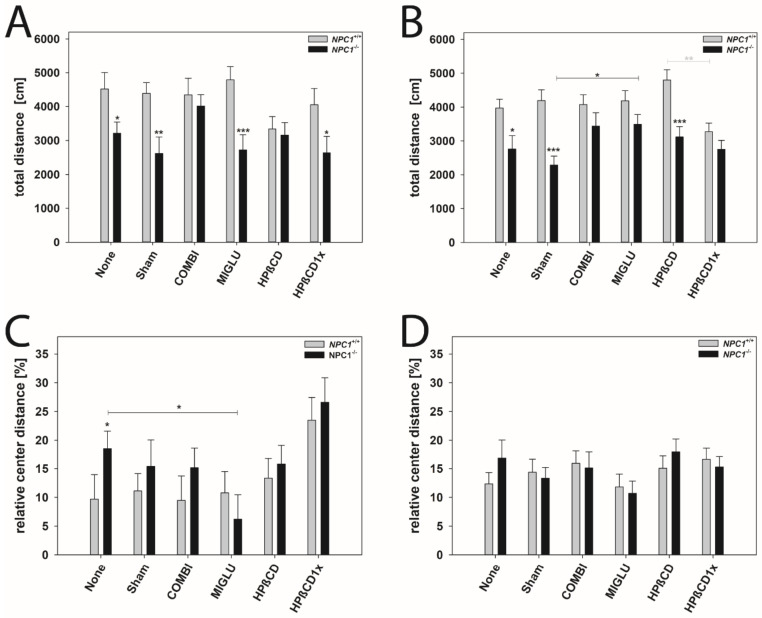
Open field test revealed differences in locomotoric activity (**A**,**B**) and anxiety-related behavior (**C**,**D**). Male (**A**,**C**) and female (**B**,**D**) *NPC1* mice. Significant post hoc-tests are indicated by asterisks (* *p* < 0.05, ** *p* < 0.01, *** *p* < 0.001). Data are means ± SEM.

The open field test was conducted to assess explorative locomotor activity and anxiety-related behavior (Figure 7A–D). Total walking distance and relative center distance were measured and compared between the various groups and genders (Figure 7A–D).

Total distance: All male *NPC1^+/+^* mice groups walked about 4241 cm in 10 min (Figure 7A). No significant intergroup differences were evaluated (Figure 7A). Male *NPC1^−/−^* mice of the None, Sham, MIGLU, and HPßCD1x groups displayed reduced motor activity (None 3214 cm, Sham 2614 cm, MIGLU 2724 cm, HPßCD1x 2636 cm) compared with the respective wild types (Figure 7A). However, male *NPC1^−/−^* mice of the COMBI and HPßCD groups (COMBI 4012 cm, HPßCD 3162 cm) nearly reached the values of the respective wild type groups (COMBI 4351 cm, HPßCD 3340 cm) (Figure 7A). All in all, neither treatment of male *NPC1^−/−^* mice significantly increased total distance compared to the None or Sham groups (Figure 7A).

All female *NPC1^+/+^* mice groups walked about 4083 cm in 10 min without any significant intergroup differences (Figure 7B). All female *NPC1^−/−^* mice in the mean walked about 2973 cm in 10 min. Female *NPC1^−/−^* mice of the None, Sham, and HPßCD groups displayed reduced motor activity (None 2762 cm, Sham 2286 cm, HPßCD 3118 cm) compared with the respective wild types (None 3974 cm, Sham 4191 cm, HPßCD 4800 cm) (Figure 7B). However, female *NPC1^−/−^* mice of the COMBI, MIGLU, and HPßCD1x groups (COMBI 3439 cm, MIGLU 3487 cm, HPßCD1x 2784 cm) nearly reached the values of the respective wild type groups (COMBI 4075 cm, MIGLU 4183 cm, HPßCD1x 3277 cm) (Figure 7B). The mean total distance travelled by the MIGLU-treated female *NPC1^−/−^* mice (3487 cm) exceeded those of the Sham-treated ones (2286 cm, *p* < 0.05). The values of the other three female *NPC1^−/−^* treatment groups, however, did not differ significantly from the respective None and Sham groups (Figure 7B).

Relative center distance: This parameter calculated the distance walked in the four central quadrants as a percentage of the whole distance walked in the complete open field and, by this, quantifies anxiety-related behavior. All in all, neither treatment of male and female *NPC1^+/+^* and *NPC1^−/−^* mice significantly altered the relative center distance compared with the respective None or Sham groups, except for decreased values of the male MIGLU-treated *NPC1^−/−^* mice compared with the respective None group (Figure 7C,D).

**Figure 8:**
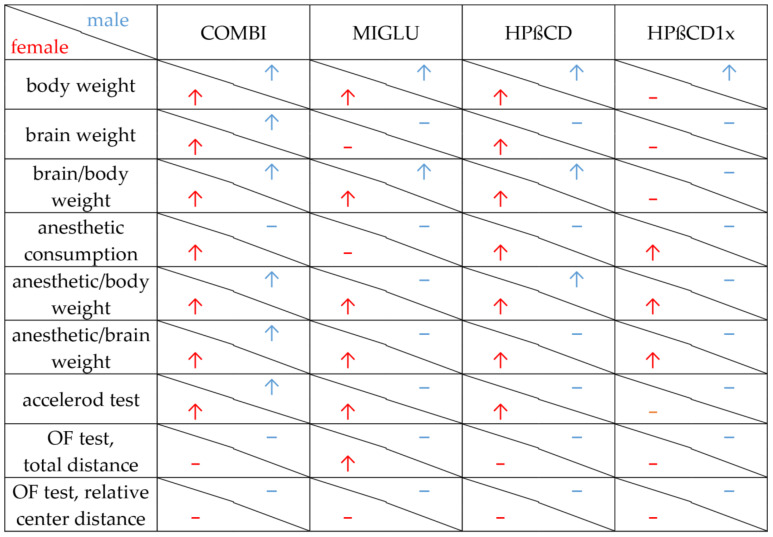
Changes, induced by COMBI, MIGLU, HPßCD and HPßCD1x in *NPC1^−/−^* mice. Significant amelioration (↑) or no significant change (−) compared with the respective None and/or Sham groups. Apart from body weight data, all other data shown here are novel.

Figure 8 summarizes the results of all parameters measured in the various treatment groups of male and female *NPC1^−/−^* mice. The respective figure depicting *NPC1^+/+^* mice is given in the Supplementary Materials (Figure S1).

## 3. Discussion

The therapeutic benefit of the COMBI (MIGLU, ALLO, HPßCD) medication in *NPC1^−/−^* mice is well described for various parameters [32,34,61,62,63,65,67,68]. Here, in one trial, in addition to COMBI treatment, we investigated the role of the single components MIGLU and HPßCD by studying 24 different groups of mice. In each case, male and female *NPC1^+/+^* and *NPC1^−/−^* mice were evaluated separately, applying six treatment strategies to each of them.

To our knowledge, up to now, most studies on the therapeutic effects of various drugs in *NPC1^−/−^* mice and the respective wild types were evaluated in gender-mixed groups [32,34,48,62,69,70,71]. Clinical observations in NPC1 patients described primarily age-dependent heterogeneity of the beginning, expression, and symptoms of the disease without differentiation of the patients’ gender [6,72,73,74,75]. There was no gender-dependent survival difference in NPC patients [76]. Interestingly, Walterfang et al. [77] described two siblings with schizophrenia whose adult NPC genotypes were identical, but showed dimorphism in illness course, clinical, and biochemical parameters. The authors suggested that female patients might have a differential illness course and degree of impairment, and sex steroids may play a role; however, they stated that human data were lacking on the effect of sex on the biochemical and clinical parameters in NPC disease.

Here, we investigated different parameters in sufficiently large cohorts of males and females separately in *NPC1^+/+^* and *NPC1^−/−^* mice including their influenceability by drugs.

### 3.1. Both Medications Prevent Body Weight Loss, Preferably in Females

In male wild type mice, we observed significantly reduced body weight due to COMBI treatment, but not due to the other treatment option. These results fit with our previous findings in wild type *NPC1^+/+^* mice [32], where the reduction of the body weight occurred after COMBI treatment, but not following MIGLU treatment. The authors explained this phenomenon as an effect of HPßCD as part of COMBI, which is probably due to an additive interaction of both molecules on the synthesis or metabolism of lipids [32,78]. Actually, administration of either MIGLU or HPßCD alone had no effect in *NPC1^+/+^* mice, but was beneficial in *NPC1^−/−^* mice.

As weight loss is a significant indicator of the progression of NPC1 disease [34,49], the diminished weight loss can be regarded as a therapeutic effect in *NPC1^−/−^* mice when compared to the respective None- or Sham-treated groups [4,16,34,71,79,80,81,82,83,84,85].

All drug-treated groups of *NPC1^−/−^* mice studied in this investigation led to body weights that were significantly higher compared with the respective None or Sham groups. These results corroborate and complement data that have already described increased body weight of *NPC1^−/−^* mice after COMBI treatment [34,62]. Although COMBI therapy restores cholesterol homeostasis in the spleen, a strongly affected organ in *NPC1^−/−^* mice [10], the special mechanism of each drug resulting in an amelioration of body weight has not yet been clarified.

The iminosugar MIGLU prevents pathologic lipid accumulation because MIGLU specifically inhibits the enzyme glucosylceramide synthase (GCS) that converts ceramide into glycosphingolipid glucosyl-ceramide (i.e., the first product in the synthesis of complex glycosphingolipids including gangliosides) [38,86,87,88]. At the same time, MIGLU has a positive influence on calcium homeostasis [4,89,90]; both mechanisms presumably prevent weight loss of *NPC1^−/−^* mice.

HPßCD works by suppressing the sterol regulatory element-binding protein-2 (SREBP2) target genes, which ultimately leads to reduced cholesterol synthesis. Excess cholesterol is shifted into the metabolically active pool and finally excreted in the bile acid, thereby decreasing the amount of pathologic lipid deposits [52]. Acute subcutaneous injection of HPßCD (4000 mg/kg body weight) rapidly overcame the transport defect in *NPC1^−/−^* mice, injected either at P7 or P49, even though this compound was cleared from the body and plasma six times faster in the mature mouse than in the neonatal animal (Liu et al. 2010). The free cholesterol flows into the cytosolic ester pool, suppresses sterol synthesis, downregulates SREBP2 and its target genes, and reduces expression of macrophage-associated inflammatory genes. These effects were seen in the liver and brain as well as in peripheral organs like the spleen and kidney. Only the lung appeared to be resistant to these effects. Forty-eight hours after HPßCD administration to 49-day-old mice, fecal acidic sterol output increased, whole-animal cholesterol burden was reduced, and the hepatic and neurological inflammation were ameliorated. Interestingly and surprisingly, in our study, the male *NPC1^−/−^* mice of the HPßCD1x group benefitted from a single injection at P7; this seemingly complements the study of Liu et al. [52]. These authors demonstrate that HPßCD administration reverses the cholesterol transport defect seen in *NPC1^−/−^* mice at various ages, and this reversal allows the sequestered sterol to be excreted from the body as bile acid [52]. However, how exactly the various therapeutic regimes and their mechanisms of action counteract the body weight could not yet be conclusively clarified and, therefore, further studies are necessary to understand the exact genesis of weight loss and to optimally align therapeutic targets.

All female wild type mice showed nearly equal body weight irrespective of treatment scheme. Moreover, as in all other parameters studied here, the various therapeutic interventions did not have a single significant effect on female wild type mice. In the None and Sham groups, the body weight of the female *NPC1^−/−^* mice showed significantly lower values than the respective wild types (Table 1). However, compared with male *NPC1^−/−^* mice, weight loss in the respective females was less pronounced, as already noticed by Võikar et al. [26] in untreated animals. Following treatment with COMBI, MIGLU, and HPßCD, body weight significantly increased compared with the None group and revealed no significant differences compared with the respective wild type groups. Thus, female *NPC1^−/−^* mice had a greater benefit from the various therapies than *NPC1^−/−^* males.

The question arises as to how the differences of *NPC1^−/−^*-related gender-specific weight reduction—males lost more weight than females—and its drug-induced prevention of weight loss—females profited relatively more from drug therapy than males—can be explained.

Generally, the body weight of mice depends mostly on sex chromosomes, sex hormones, and the firing rate of pro-opiomelanocortin (POMC)-expressing hypothalamic neurons [91,92,93,94,95,96]. Two studies determined testosterone concentrations in untreated male *NPC1^−/−^* mice [97,98]. According to Akrovi et al. [97], circulating testosterone levels in *NPC1^−/−^* males were significantly decreased to one-third of the wild type value: *NPC1^+/+^* mice showed 334.07 ± 42.50 pg/mL and *NPC1^−/−^* mice 109.93 ± 15.88 pg/mL. Comparable values were revealed by Roff et al. [98]: *NPC1^+/+^* mice 482 ± 131 ng/dl and *NPC1^−/−^* mice 70 ± 21 ng/dl.

In females, plasma concentrations of progesterone were not significantly different between *NPC1^−/−^* and *NPC1^+/+^* mice [99,100,101]. The progesterone content in the brains of wild type mice was about 2 ng/mg protein and in the *NPC1^−/−^* group about 1.7 ng/mg protein. Estradiol levels, however, were reduced in the brains and ovaries of *NPC1^−/−^* mice [101,102]. The content of estradiol in the brains of the wild type mice was about 2.4 ng/mg protein and in the *NPC1^−/−^* group, it was 1.7 ng/mg protein, respectively [101]. Using a heterotypic neuron-glia co-culture system, Chen et al. [101] found that estradiol content was decreased both in pure *NPC1^−/−^* astrocyte culture medium and in *NPC1^−/−^* mouse brain: estradiol content in astrocyte culture medium from wild type mice was about 220 pg/ng and about 150 pg/ng from the *NPC1^−/−^* group.

Apparently, the more pronounced deficits in the sex hormones of male *NPC1^−/−^* mice could account for their more pronounced weight loss compared to the untreated female None and Sham groups. Unfortunately, as yet, no data on hormone concentrations are available for our other experimental groups testing the hypothesis of a possible, at least partly drug-induced normalization of the respective hormones in *NPC1^−/−^* mice.

### 3.2. There Is No Drug-Related Effect on Brain Weights

Generally, brain weight in both sexes of *NPC1^−/−^* mice was less dramatically reduced than body weight and therefore less increased in the various therapeutic groups.

In male *NPC1^−/−^* mice, COMBI and HPßCD treatment significantly increased brain weight compared to the None group. Nevertheless, irrespective of treatment, *NPC1^−/−^* mice still had lower brain weights than the respective wild type mice.

In female *NPC1^−/−^* mice, we found increased brain weights in the COMBI- and HPßCD-treatment groups und unchanged brain weights in the MIGLU and HPßCD1x groups compared with the respective None or Sham group. As in male *NPC1^−/−^* mice, irrespective of treatment, female *NPC1^−/−^* mice still showed lower brain weights than the respective wild type mice. Seemingly, HPßCD could positively change yet unknown parameters that positively influence brain development. Nevertheless, after COMBI treatment, lipid analyses in various regions of *NPC1^−/−^* mice showed disrupted sphingosine-1-phosphate lipid (S1P) metabolism in all brain regions, together with distinct changes in S1pr3/S1PR3 expression [103]. Interestingly, brain regions of *NPC1^−/−^* mice showed only weak COMBI-treatment effects in these parameters [103]. As those lipids showed only weak COMBI-treatment effects in *NPC1^−/−^* mouse brains [103], the mostly absent positive treatment effect on brain weight seemed plausible.

Generally, brain weight depends on the amount and size of neurons and glia cells including myelination, and the neuropil [104,105,106].

*NPC1^−/−^* micehad lower brain weights than the respective wild type mice. These findings correlate with other data on the reduction of brain weight in NPC disease [61,71,107]. Significant volume reductions in the cerebellar hemispheres, the medulla oblongata, the corpus callosum, and the olfactory bulb of *NPC1^−/−^* mice have been described [68,108,109,110], which could contribute to the difference in brain weight [24,109]. A further example at the terminal stages in the murine NPC1 model is an age-dependent Purkinje cell loss of 96% [22,34,107,109]. Maass et al. [61] confirmed Purkinje cell degeneration (83%) in lobe VIII of *NPC1^−/−^* mice. In addition, a 44% reduction of molecular layer interneurons and a 16% reduction of Golgi interneurons were calculated in the respective cerebellar cortex [61]. Furthermore, a significant decrease in volume and a reduction in cell counts in the medial and the lateral cerebellar nuclei in *NPC1^−/−^* mice were found. As shown by others, the Purkinje cell loss at the terminal stages in the NPC1 model could be partially prevented by a COMBI treatment [22,34,109,111]. We also found that COMBI-treated *NPC1^−/−^* mice revealed an increase in Purkinje cell numbers, which reached about 71% Purkinje cells compared with wild type mice in lobe VIII [61]. Moreover, a full positive therapeutic effect on the volume of the molecular layer and on the cell count of molecular interneurons was observed [61].

Intracellular ganglioside stores and neuronal degeneration were shown in large pyramidal and Purkinje cells, and in neurons of the thalamus and the hippocampus [39,109,112,113]. In particular, hypomyelination was conspicuous in the cerebral white matter and corpus callosum of *NPC1^−/−^* mice and surely is one basis for their lower brain weight [114,115,116,117,118]. In *NPC1^−/−^* mice, from early postnatal development onwards, defects of differentiation of post-mitotic premyelinating oligodendrocytes into mature myelinating oligodendrocytes have been described by various groups [114,117,118]. However, the amount of demyelinization varies between different parts of the brain [119,120,121].

We found that male wild type mice of the COMBI group had significantly reduced brain weights compared with the None or Sham groups, whereas brain weights of the MIGLU, HPßCD, and HPßCD1x groups were unchanged. These results are in the line with our former results in wild type mice treated with COMBI [32]. The smaller brains of the COMBI group could most likely be a consequence of their reduced body weight [32].

Additionally, female *NPC1^+/+^* mice of the COMBI group had significantly reduced brain weights compared to the None and Sham groups. At the same time, brain weights of the MIGLU, HPßCD, and HPßCD1x groups were unchanged compared to the None and Sham groups. In females, smaller brain weight of the COMBI group could be a consequence of the reduced body weight.

Importantly, in both male and female wild type mice, COMBI treatment caused a significant reduction in brain weight. Our hypothesis that COMBI treatment in wild type mice results in concentrations of cholesterol and other lipids below or above normal levels, interfering with normal cellular or membrane functions, was substantiated by Gläser et al. [103]. They showed that COMBI treatment of *NPC1^+/+^* mice resulted in strongly decreased S1P amounts in all brain regions investigated [103]. Besides S1P, other lipids showed COMBI-induced changes such as a strong increase of monohexosyl ceramides (MC) 18:0 and monohexosyl dihydroceramides (MDC) 18:0, and ceramides (Cer) 24:1 and Cer 26:1 in some brain regions, or a decrease of lactosylceramides (LC) 16:0 and LC 22:0 in the hippocampus. Seemingly, all other treatment options did not change these parameters in wild types. This, however, has to be determined in future experiments.

### 3.3. Brain Weight/Body Weight Ratio

Body weights of male mice exceeded those of females. At the same time, brain weights of females and males were in a similar range. As in the drug-treated *NPC1^−/−^* mice, body weight nearly normalized, and simultaneously, the respective brain weight only marginally increased; brain weight/body weight ratios became constant in nearly all treated groups of *NPC1^−/−^* and wild type mice. However, all females had larger brain weight/body weight ratios due to their lower body weight compared with males.

### 3.4. Female NPC1^−/−^ Mice Need More Anesthetic Drugs Than Males

All mutant *NPC1^−/−^* mice in both genders needed more anesthetic solution compared with wild types, when the ratio of anesthetic solution per brain weight was considered. Interestingly, in contrast to males, female *NPC1^−/−^* mice of the None and Sham groups needed significantly more drug mixture compared to the treated groups. Altogether, although there was a clear treatment effect in *NPC1^−/−^* females, all groups still needed significantly higher amounts of drug mixture compared with the corresponding wild types.

However, *NPC1^+/+^* mice of both genders needed almost equal dosages of anesthetic solution. In contrast, None- and Sham-treated female *NPC1^+/+^* needed less anesthetic solution per brain weight compared with the respective wild type males. Information about gender differences in the dose of anesthetic management in patients with Niemann-Pick disease has not yet been reported. Only possible complications of anesthetic management and the necessary measures in NPC patients have been shown [122,123,124,125,126,127,128] and explained by the disease-related interference with pathologies of liver, spleen, lung, and heart [129]. Due to both the liver involvement and the probability of an alteration in drug metabolism, anesthetic drugs should be used with caution in NPC patients [122].

Ketamine has potent actions on NMDA receptors, and less potent actions on sigma 1 receptors; it also binds with even lower affinity to µ opiate receptors as well as to the transporters for norepinephrine and serotonin and has been shown to decrease depressive symptoms in humans [130,131,132]. Xylazine (*Rompun^®^*) (Bayer Animal Health, Leverkusen, Germany) is a strong α2-adrenergic agonist, the effects of which are mediated via stimulation of central α2-receptors. α2-adrenergic stimulation decreases the release of norepinephrine and dopamine in the central nervous system resulting in sedation, muscle relaxation, and decreased perception of painful stimuli [133].

It can be speculated that the gender-specific differences of wild types in sensitivity to anesthetics could be due to differences in transmitter concentrations or their respective receptors in the brain [134,135,136,137,138,139].

We found that *NPC1^−/−^* mice of the None and Sham groups in both genders, and especially females, needed as much or often more drug mixture than the respective wild type group, although their body weight was considerably lower. Normal female rats [140,141,142] and female mice [133,138,143] have been found to be more sensitive to ketamine and showed sensitivity to lower doses of the drug to which the males did not react [141]. The difference in this sensitivity has been discussed to be most likely gonadal hormone-dependent. In this line, the rapid antidepressant-like effects of a low dose of ketamine that was effective only in naïve female rats, were completely abolished in ovariectomized rats, but emerged again upon restoration of physiological levels of estrogen and progesterone [142]. These data suggest an important role for sex hormones in enhancing the antidepressant-like effects of lower doses of ketamine in the females, but the innate mechanisms pertaining to these effects are still elusive [143].

The *NPC1* gene is important for the normal development of reproductive functions and this is illustrated by the fact that male and female *NPC1^−/−^* mice are sterile and display important histologic abnormalities in their gonads [63,97,102]. Moreover, studies have shown that although plasma concentrations of progesterone were not significantly different between *NPC1^+/+^* and *NPC1^−/−^* females [99,100], estradiol levels were reduced in the brain and ovaries of these mice [101,102]. Therefore, we hypothesize that NPC1 deficiency could affect the synthesis and plasma concentrations of sex steroid hormones and subsequently influence metabolism through them, and probably also the sensitivity of the female *NPC1^−/−^* mice to ketamine. In female *NPC1^−/−^*, the effect of treatment in reducing the dosage of anesthetics is significant; particularly the COMBI and HPßCD treatment in female *NPC1^−/−^* mice was highly effective in lowering the needed dose. As up to now, no knowledge about transmitters and their receptors or changes in hormonal status in female *NPC1^−/−^* mice with various therapies exists, a final explanation for the positive drug effects is not available.

### 3.5. Accelerod Test—Motoric Coordination in NPC1^−/−^ Mice Is Improved by All Drugs

The rotarod and accelerod test performance were used to measure motor coordination, skill learning, balance, and ataxia [144,145]. During the rotarod test phase, all mice learnt the task during training trials 1–8; the worst training effects were seen in the None and Sham groups of *NPC1^−/−^* mice of both genders. In neither gender, a significant drug effect on accelerod performance of *NPC1^+/+^* mice was observed. In both genders, None and Sham groups of *NPC1^−/−^* mice performed more poorly than their respective wild types. In male *NPC1^−/−^* mice, COMBI treatment improved test results. However, female *NPC1^−/−^* mice significantly benefitted from COMBI, MIGLU, and HPßCD treatment. Comparison of the accelerod of both genders revealed that female *NPC1^+/+^* mice of the MIGLU and the HPßCD groups performed better than the respective males. In *NPC1^−/−^* mice gender-specific differences were only seen in MIGLU-treated animals: females performed significantly better than the respective males.

Hovakimyan et al. [62] have already shown that the COMBI-treated *NPC1^−/−^* mice of both genders demonstrated a significantly better accelerod test performance when compared to the Sham-treated mutant mice, corroborating the present results. Here, also HPßCD and MIGLU groups of female *NPC1^−/−^* mice reached an accelerod performance comparable to that of wild type mice. The positive effects of MIGLU and HPßCD, especially in female *NPC1^−/−^* mice, could be due to drug-induced changes in lipid metabolism and hormone concentrations [10,63,97,102,103]. The drugs improved accelerod performance in *NPC1^−/−^* mice. At the same time, no drug caused alteration of motor coordination and balance in comparison with the respective Sham-treated *NPC1^+/+^* animals, confirming the data of Schlegel et al. [32] for MIGLU and COMBI treatment [32]. This speaks in favor of the hypothesis that the drugs used improve *NPC1^−/−^*-related pathologic lipid metabolism and only exert a minor influence on the normal status [10,34,51,63,103].

The mutant female mice of the MIGLU group performed significantly better than those of the male groups, which corresponds to results of Võikar et al. [26]. These authors applied a behavioral test in order to establish the onset and development of the major symptoms in the NPC1 Balb/C mouse model including female and male mice groups, and showed that the female *NPC1^−/−^* mice appeared to preserve their motor coordination abilities better and also longer than the male *NPC1^−/−^* mice. They explained these results with a number of other reports on retained neurological functions and/or neuroanatomy in female mutant mice with the cerebellar behavioral phenotype [146,147,148]. This raises the intriguing possibility that the male mice may be more vulnerable to *NPC1^−/−^*-related factors that disrupt motor coordination. Another reason why female *NPC1^−/−^* mice of the MIGLU group performed significantly better than male groups is probably the increased drug-induced changes in sex steroid hormone levels [26].

*NPC1^−/−^* mice of both genders of the HPßCD1x groups performed as badly as the None or Sham groups in the accelerod tests. This corroborates the observation of Liu et al. [51] in *NPC1^−/−^* mice after a single administration of HPßCD at P7. One single administration of HPßCD at P7 generally is not without any positive effect, which is reflected by other results of the present study: the body weights of the male *NPC1^−/−^* mouse HPßCD1x group increased compared to the respective None and Sham groups; in the female *NPC1^−/−^* mice of the HPßCD1x group, anesthetic consumption was reduced compared to the None and Sham groups.

### 3.6. Open Field Test—No Apparent Improvements upon Treatment

Spontaneous horizontal locomotor activity, anxiety, and unconditioned exploration were estimated via the OF test [149,150,151]. Here, the total walking distance and relative center distance were measured.

All female and male *NPC1*^+/+^ mice of the twelve experimental groups walked about the same distance in 10 min. In both genders of NPC1*^−/−^* mice, the None and Sham groups walked shorter distances compared with the respective wild types, and most groups of treated *NPC1^−/−^* mice displayed reduced motor activity, which did, however, reach a significant level in less than half of the groups. Significantly improved total walking distances compared with the Sham group was only found in the MIGLU-treated female *NPC1^−/−^* mice, so this parameter turned out not to be a specifically differencing parameter to show drug treatment effects on *NPC1^−/−^* mice.

We also measured the relative center distance as an indicator of anxiety-related behavior. All wild type mice investigated showed nearly the same relative center distances and, thus, comparable anxiety. Comparison of the respective female and male Sham, COMBI, MIGLU, HPßCD, and HPßCD1x groups revealed no significant differences in the relative center distances between *NPC1*^+/+^ and *NPC1^−/−^* mice. Interestingly, comparison of the female and male None groups of the wild type and mutant mice revealed that male *NPC1^−/−^* mice were less anxious than the untreated wild type mice group; however, the respective females showed no significant difference.

The present results corroborate former ones that there was no evidence for changed anxiety in the Sham-treated *NPC1^−/−^* mice when compared with the respective controls as well as with COMBI-treated mutant mice [62]. Additionally, Schlegel et al. [32] observed no significant differences in the ratio of center to total visits between Sham-, MIGLU-, and COMBI-treated wild mice and, therefore, both COMBI and MIGLU therapy leave the motor capabilities and spontaneous motor behavior of *NPC1*^+/+^ mice unaltered.

Yadid et al. [152] explored the relationship between neurological damage and neurochemical changes in the brains of *NPC1^−/−^* mice. Their results revealed significant changes in the neurotransmitter levels in the cortex and the cerebellum of mutant mice [152]. In particular, they described an increased content of GABA in the cortex of mutants, whereas serotonin levels were reduced. As GABA and serotonin are transmitters that are important in the regulation of motor and emotional behavior [153], it can be speculated that their altered concentration in mutant mice could be correlated to the reduced total distance travelled in the mutants of the None and Sham groups.

## 4. Materials and Methods 

### 4.1. Animals

All animal procedures were approved by the local authorities (Landesamt für Landwirtschaft, Lebensmittelsicherheit und Fischerei des Landes Mecklenburg-Vorpommern; approval ID: 7221.3-1.1-030/12, 14 June 2012). All institutional guidelines for animal welfare and all experimental conducts were followed and all efforts were made to minimize suffering.

Heterozygous *NPC1^+^*^/-^ mice breeding pairs of NPC1 mice (BALB/cNctr-Npc1^m1N^/-J) were obtained from Jackson Laboratories (Bar Harbor, ME, USA) to generate homozygous *NPC1*^−/−^ mutants and *NPC1*^+/+^ control wild type mice. Mice were maintained under standard conditions with free access to food and water with a 12 h day/night cycle, a temperature of 22 °C and a relative humidity of about 60%. Genotypes were determined up until postnatal day P7 by PCR analysis of tail DNA [62,67]. *NPC1*^−/−^ mutants and *NPC1*^+/+^ wild type controls of both sexes were used for different therapeutic treatment schedules. Altogether, 122 control (66 female, 56 male) and 117 mutant mice (62 female, 55 male) were involved in this study. The exact numbers of animals investigated in the various groups were (n total group, n females, n males): None *NPC1^+/+^* 20, 14, 6; Sham *NPC1^+/+^* 22, 9, 13; COMBI *NPC1^+/+^* 20, 12, 8; MIGLU *NPC1^+/+^* 20, 10, 10; HPßCD *NPC1^+/+^* 20, 11, 9; HPßCD 1x *NPC1^+/+^* 21, 16, 5; None *NPC1^−/−^* 17, 6, 11; Sham *NPC1^−/−^* 19, 13, 6; COMBI *NPC1^−/−^* 20, 6, 14; MIGLU *NPC1^−/−^* 20, 12, 8; HPßCD *NPC1^−/−^* 21, 12, 9; HPßCD 1x *NPC1^−/−^* 20, 14, 6.

### 4.2. Treatment

**Figure 9:**
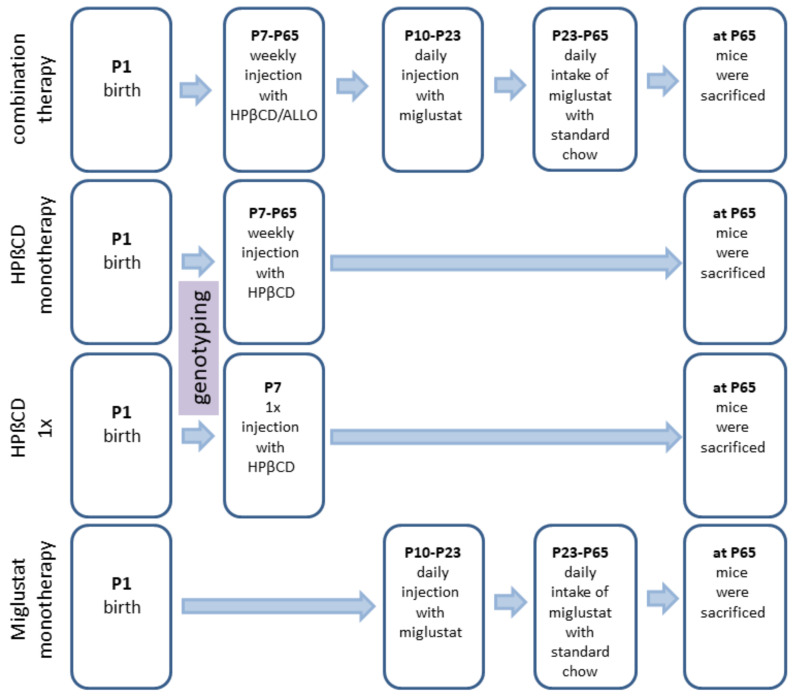
Timeline of drug administrations for all experimental groups. Mice of the Sham groups were injected with the respective amounts of 0.9% NaCl according to the treatment plan of the combination-treated group. At P7 and thenceforth, NPC1 mice were injected weekly with allopregnanolone (25 mg/kg; Sigma Aldrich, St. Louis, MO, USA) dissolved in HPßCD (4000 mg/kg; Sigma Aldrich, Munich, Germany). At P10 and until P23, animals were injected daily with miglustat (300 mg/kg, i.p.; Zavesca; Actelion Pharmaceuticals, San Francisco, CA, USA). From P23 onward, animals were fed with miglustat included in standard chow (1200 mg/kg per day) until termination.

We investigated 24 different groups: *NPC1^−/−^* mice (mutant mice) treated with (i) no therapy (None), (ii) vehicle injection (Sham), (iii) combination of MIGLU, ALLO and HPßCD (COMBI), (iv) MIGLU alone (MIGLU), (v) HPßCD alone starting at P7 and repeated weekly throughout life (HPßCD), and (vi) HPßCD alone given only once at P7 (HPßCD1x). The six respective *NPC1^+/+^* mice (wild type mice) groups were evaluated in parallel. In order to look for possible gender-specific drug effects, groups of male and female mice were studied separately (Figure 9).

Combination therapy (COMBI group): Starting at postnatal day 7 (P7) and weekly thereafter, mice were injected with HPßCD/ALLO (25 mg/kg ALLO dissolved in 40% HPßCD) (both from Sigma-Aldrich, Munich, Germany). Additionally, from P10 until P23, mice were injected daily with MIGLU (300 mg/kg, i.p.; Zavesca; Actelion Pharmaceuticals, San Francisco, CA, USA), dissolved in saline. Starting at P23 and until termination of the experiments, mice were fed standard chow (V1184-000, Ssniff, Soest, Germany) including MIGLU at a daily dose of 1200 mg/kg. 

HPßCD monotherapy (HPßCD group): HPßCD was injected starting at postnatal day 7 (P7) and weekly thereafter, in the same amount as included in COMBI (4000 mg/kg, i.p.; Sigma Aldrich, Munich, Germany).

HPßCD1x (HPßCD1x group): These mice received only a single injection of HPßCD at P7 (4000 mg/kg, i.p.).

MIGLU monotherapy (MIGLU group): Comparable with COMBI, mice were injected daily with MIGLU (300 mg/kg, i.p.) at P10 until P23. From P23 onward, animals were fed standard chow (V1184-000, Ssniff, Soest, Germany) including MIGLU at a daily dose of 1200 mg/kg.

Sham (Sham group): Sham-treated mice were injected following the scheme of the COMBI mice, however, omitting the drugs in the saline.

None (None group): These mice were not injected at all.

All mice were sacrificed after the open field test at P65.

### 4.3. Body Weight and Brain Weight

Body weight as well as the dosage of drugs inducing anesthesia were estimated prior to sacrifice. Brain weight was determined as fresh weight or weight after perfusion fixation. Both weights did not differ significantly.

### 4.4. Anesthesia Consumption

Before scarification, animals were deeply anesthetized with a house-made drug mixture, a diluted solution of 0.75 g ketamine hydrochloride (contained in 7.5 mL of a 10% ketamine hydrochloride ready to use preparation; Ketamin^®^ 10%, Bela-Pharm, Vechta, Germany) + 0.05 g xylazine (contained in 2.5 mL of a 2% xylazine ready to use preparation; Rompun^®^, Bayer, Leverkusen, Germany), and 90 mL saline. Mice were given dosages in 0.1 mL steps of drug mixture sufficient to remove the between toes reflex. A wild type male mouse of about 25 g body weight needed about 0.35 mL of drug mixture, containing 1.75 mg ketamine hydrochloride and 0.12 mg xylazine.

### 4.5. Accelerod Test

The motor coordination, balance, and ataxia of mice were evaluated using accelerod (accelerated rotarod) performance on P60–P63 (Jones und Roberts 1968). The accelerod test has been shown to be more sensitive than the rotarod test in detecting motor function deficits and in providing more consistent results [3]; it also enables testing of the motivation and motor learning behavior of mice [145,154]. The accelerod system for mice (TSE Systems, Bad Homburg, Germany) consists of a base platform and a rotating rod (3 cm diameter, 11.4 cm width) with a non-skid surface. One hour before starting the test, the animals were kept in the examination room to become familiar with the novel condition. For training, each mouse received four test trials per day at a constant 12 rpm for 2 min on two consecutive days. During training, the latency of the first fall down and the number of fall downs during the training trials were recorded. Animals that had fallen down were put back on the roller until the trial period was over.

In the acceleration modus, the rotation increased from 4 to 40 rpm in 30 s steps within 5 min. Each mouse received four trials per day on two consecutive days. In contrast to training, the animals were not put back on the roller once they had fallen. During the accelerod test trials, the latency and rpm reached were recorded as during the training procedure.

### 4.6. Open Field Test

Spontaneous horizontal locomotor activity and anxiety were estimated on P65 via the open field test (OF) as originally described [149,150]. One hour before starting the test, the animals were kept in the dark-phase in the examination room to become familiar with the novel condition. For the test, the mice were placed in a novel environment inside of an isolation box (TSE-Systems, Bad Homburg, Germany) with a square open field arena of 50 cm × 50 cm. The OF was divided into 16 quadratic subfields in 12 peripheral and four central areas by a grid in the tracking software. Mice were not previously habituated to the locomotor activity chamber. In a 10-min trial, the mice were subjected to a novel environment from which escape was prevented by surrounding walls. Illumination of the open field was provided by a white photo bulb providing 100 Lux (Crawley 1985). Environmental odors were removed by thorough cleaning of the open field after each session to avoid the influence of odor trails on the behavior. The movements were recorded by a video camera placed inside the isolation box and tracked using the VideoMot2 Software (TSE Systems, Bad Homburg, Germany). The total distance and the center distance traveled by the mice in the open field were analyzed [155,156]. In total, eight mice were excluded because of inactivity.

### 4.7. Data Analysis

The results are presented as means ± SEM. In all cases, *p* values ≤ 0.05 were considered significant. All data were subjected to three- or two-way ANOVA. The Holm–Sidak approach was used for post-hoc comparisons. All statistical analyses were done using SigmaPlot 14 Software (Systat Software, Inc., San Jose, CA 95110, USA).

## 5. Conclusions

Our body and brain weight data and behavioral tests showed that each drug has the potential to improve the body and brain weight loss and motoric phenotype of *NPC1^−/−^* mice. Gender-specific effects were noted, in particular, female *NPC1^−/−^* mice benefited from drug administration. 

However, COMBI therapy was found to be more effective than either drug alone. In general, more anesthetic solution was required to anesthetize *NPC1*^−/−^ mice. The increased anesthetic consumption was improved only in female *NPC1*^−/−^ mice by COMBI treatment or HPßCD monotherapy, but not by MIGLU alone. Although general locomotor activity was reduced in *NPC1*^−/−^ mice, anxiety-related behavior was not altered by the drugs. 

In summary, all treatment options present some balanced advantage/disadvantage effects for specific parameters studied. Currently, a monotherapy with intrathecally applied HPßCD seems to be the most promising, although the outcomes of several long-term phase 2/3 trials are partly controversial, due to the extremely low prevalence [157,158,159]. Results of a current multinational phase 2b/3 clinical study involving 51 patients treated with 200 mg/kg intrathecally applied HPβCD every two weeks indicate doubts that HPβCD achieves benefits when compared to a placebo [160,161]. The outcome of an open-label stage of the trial involving seven patients, sponsored by Vtesse Inc., Gaithersburg, Maryland, USA is expected for December 2021 [160].

Despite the difficulties in obtaining sufficient data for approval of HPßCD treatment in humans, gender effects should be kept in mind in the future.

## Figures and Tables

**Table 1:** Mean values ± SEM of the evaluated parameters of untreated (None groups) male and female *NPC1^+/+^* and *NPC1^−/−^* mice.

Parameter	Male		Female	
	*NPC1* ^+/+^	*NPC1* ^−/−^	*NPC1* ^+/+^	*NPC1* ^−/−^
body weight [g]	24.643	15.833 ^AAA^	20.067 ^CCC^	13.375 ^BBB D^
	±0.876	±0.669	±0.614	±0.840
brain weight [g]	0.474	0.385 ^AAA^	0.463	0.370 ^BBB^
	±0.009	±0.006	±0.006	±0.008
brain/body weight [%]	1.812	2.434 ^AAA^	2.325 ^CCC^	2.816 ^BBB DD^
	±0.117	±0.079	±0.065	±0.088
anesthetic consumption [mL]	0.414	0.617 ^AA^	0.320 ^CC^	0.725 ^BBB^
	±0.055	±0.042	±0.030	±0.040
anesthetic/body weight [mL/g]	0.019	0.039 ^AAA^	0.017	0.054 ^BBB DDD^
	±0.003	±0.002	±0.002	±0.002
anesthetic/brain weight [mL/g]	0.930	1.506 ^AAA^	0.714 ^C^	1.855 ^BBB^
	±0.136	±0.092	±0.077	±0.105
accelerod test [rpm]	16.563	9.615 ^AA^	19.214	10.982 ^BBB^
	±1.821	±1.345	±1.192	±1.821
open field test,	4519.48	3214.20 ^A^	3974.49	2762.27 ^B^
total distance [cm]	±478.04	±328.36	±258.00	±394.11
open field test,	8.20	17.80 ^A^	12.80	18,70
relative center distance [%]	±3.21	±2.16	±2.23	±3.41

^A^ Significant difference between male *NPC1*^+/+^ and male *NPC1*^−/−^, ^B^ significant difference between female *NPC1*^+/+^ and female *NPC1*^−/−^, ^C^ significant difference between male *NPC1*^+/+^ and female *NPC1*^+/+^, ^D^ significant difference between male *NPC1*^−/−^ and female *NPC1*^−/−^ (^x^ *p* < 0.05, ^xx^ *p* < 0.01, ^xxx^ *p* < 0.001).

## Data Availability

Not applicable.

## Supplementary Materials

Supplementary Materials can be found at https://www.mdpi.com/1422-0067/22/5/2539/s1.

## Abbreviations

ALLO	Allopregnanolone
COMBI	Combination therapy
Cer	Ceramides
GABA	γ-aminobutyric acid
GCS	Glucosylceramide synthase
HPßCD	2-hydroxypropyl-ß-cyclodextrin
LC	Lactosylceramides
MIGLU	Miglustat
MC	Monohexosyl ceramides
MDC	Monohexosyl dihydroceramides
NMDA	N-methyl-D-aspartate receptor
NPC1	Niemann-Pick disease type C1
Npc1	NPC1 gene
NPC1	NPC1 protein
OF	Open field test
OLs	Mature myelinatinggoligodendrocates
POMC	Pro-opiomelanocortin neurons
PSD95	Postsynaptic density protein 95
SREBP2	Sterol regulatory element-binding protein-2
S1P	Sphingosine-1-phosphate lipid
