# Supplementary material for: Gender-Specific Effects of Two Treatment Strategies in a Mouse Model of Niemann-Pick Disease Type C1
Source: Int J Mol Sci. 2021 Mar 3;22(5):2539. doi: 10.3390/ijms22052539 (PMC7962008; doi:10.3390/ijms22052539)
Supplement: Supplementary file 1 [file ijms-22-02539-s001.zip › Supplement-Table 2.docx]

**Supplementary Table 2.** Overview of results of behavioural tests of *NPC1* mice treated with various drugs.

| **Citation** | **Mice Strain, gender** | **Treatment** | **Behavior Test** | **Results** |
| --- | --- | --- | --- | --- |
| Ahmad et al. 2005 [1] | Npc1^NIH^ mutant (*NPC1^–/–^*^)^ mice on the BALB/cJ background or a mixed BALB/cJ x FVB background with the mdr1a knockout. | ALLO (Sigma, St. Louis, MO) was dissolved in a 20% solution of HPßCD in water at 1.25 mg/ml injected at 25 mg/kg subcutaneously at day 7 and intraperitoneally at later times. The *NPC1^–/–^* mice were tail tipped and genotyped at 21 days of age. *NPC1^–/–^* pups and their littermates had been injected on day 7 as described above. In the case of repeated injections, only the *NPC1^–/–^* mice were again injected on day 21 and at 2-week intervals thereafter until death. | Each mouse was evaluated weekly on a Rota-Rod test instrument. The mouse was given three trials on the Rota-Rod, and the maximal trial time was recorded. Mice that could not remain on the Rota-Rod for at least 10 sec were considered to have failed. | Rota-Rod performance was moderately enhanced until about 10 weeks in the day-7-treated mice, but gradually decayed at older ages. The double-mutant *NPC1^–/–^*, mdr1a^–/–^ single-injected mice were not significantly different from the *NPC1^–/–^* single-injected mice. |
| Alvarez et al. 2008 [2] | BALB/c mice carrying a heterozygous mutation in the *npc1* | Mice received daily intraperitoneal injections of imatinib mesylate from postnatal day (p) 7 or 28. Control groups (wild type and *NPC1* mice*)* received daily intraperitoneal injections of 0.9% NaCl. | Locomotor coordination was evaluated weekly by two tests during imatinib treatment. In the hanging test, the mouse was placed to hang at the center of a horizontal bar (3 mm diameter; 35 mm long) with forepaws. The body position of the animal was observed for 30 s and scored as described in Voĩkar et al. (2002). In the beam test mice were placed at the end of a beam (100 cm long; 2.5 cm wide). Animals were trained to finish the task as quickly as possible. The number of falls during the test was counted. Footprint analysis was performed in imatinib-treated animals from p28 at 8 wk of age. | Imatinib therapy improves locomotor function in *NPC 1* mice:  Coordination and locomotor function was evaluated at regular intervals throughout treatment. The imatinib-treated *NPC1* mice exhibited a significant improvement in the hanging test, which evaluates coordination of the four paws and the tail. A delay in the onset of symptoms was observed in p28 and p7 imatinib-treated mice from 7 and 8 wk, respectively. In the beam test, which evaluates equilibrium and coordination, the imatinib-treated *NPC1* mice showed fewer falls than untreated *NPC1* mice. In this test, improvement was observed from 6 wk for both p7 and p28 imatinib-treated mice. In footprint pattern test, imatinib-treated *NPC1* mice showed a significant improvement compared with untreated *NPC1* mice. Although a significant difference in locomotor function between *NPC1* mice receiving imatinib treatment from p7 and p28 was not found, there was an increase in survival of mice in the p7 imatinib-treated group. |
| Bascunan-Castill et al. 2004 [3] | *Npc1*^NIH^ mutant mice from the BALB/cJ background | Two different drugs were investigated as a treatment in disease progression. Tamoxifen was prepared by dissolving 16 µg of the drug per 1 ml of peanut oil at room temperature (23°C). Peanut oil was used as a control. D-alpha tocopherol (brand “Natural Liquid Vitamin E”), was purchased from Solgar.  Treatment was initiated at day 21. The mice were divided into four groups: control, tamoxifen treated, vitamin E treated, and tamoxifen plus vitamin E treated. | Each mouse was evaluated weekly on a Rota-Rod test instrument. The Rota-Rod was driven at a constant rate of 25 rpm. The mouse was given 3 trials on the Rota-Rod, and the maximum trial time was recorded. Mice that could not remain on the Rota-Rod for 10 seconds were considered to have failed. | Control mice showed no significant sex differences (58 days, 56-61 days 95% CI). Among tamoxifen-treated mice, there was a slight delay before failure on the Rota-Rod (64 days, 51-71 days 95% CI). Mice treated with vitamin E showed a 7-day improvement over controls, with a median failure age of 65 days (62–66 days, 95% CI). Mice treated with vitamin E were not significantly different from mice treated with tamoxifen. Mice given both treatments, showed an 11-day increase over controls with a median failure age of 69 days (58-74 days, 95% CI). In addition, these mice showed a significant increase in absolute Rota-Rod performance (time spent on rotating rod) in the early stages of disease progression. |
| Borbon et al. 2012 [4] | *NPC^−/−^* mice were obtained through breeding heterozygous *NPC1^+/−^* mice which are maintained on the BALB/cJ background. | A total of five diets consisting of three concentrations of curcumin and controls were tested: 1) Chromadex, 2% curcumin mixed in NIH 7013 chow; 2) Acros, 0.17% curcumin mixed in 5P14 base chow; 3) NIH 7013, chow alone; 4) Lipidated curcumin delivered at 0.25% in NIH 7013 chow and 5) Lipidated vehicle, | In order to evaluate balance and motor coordination, mice were tested using two different tests, the balance beam and the coat hanger. | The results from the balance beam show that *NPC1^+/+^,* wild type control mice and *NPC1^−/−^* mice on the 2% curcumin (Chromadex) diet performed quite similarly. Mice might show improvements in motor behavior and/or memory and yet not have greatly increased survival. The time spent on the beam was not different. The *NPC1^−/−^* untreated and the *NPC1^−/−^* mice on the Acros diet were similar and had lower scores. The results from the coat hanger test show a decline in score (performance rating) over age for NPC1^−/−^ mice untreated or treated with either diet, as opposed to a constant high score for NPC1^+/+^ control mice. The high dose (Chromadex) that improved balance beam performance, showed a non-significant improvement by 9 weeks which again could be important for morbidity. |
| Chen et al. 2007 [5] | A breeding pair of BALB/c NPC1NIH mice were obtained from Jackson Laboratories | Estradiol Treatment:  *NPC1^-/-^* mice received subcutaneous injections of 2 mg/kg or 20 mg/kg of 17b-estradiol in corn oil weekly from P7; the concentrations of 17b-estradiol were 10 ng/ 10 lL or 100 ng/10 lL. | Rota-Rod Performance Test:  The apparatus was turned on at the minimum speed, the mice were positioned on the rotating rod, and acceleration started immediately until 10 rpm was reached. Three testing sessions were performed at 5 min intervals. Results were expressed as the longest time that a mouse remained on the treadmill. Mice were tested every 5 days from P30. | Estradiol treatment improved behavioral deficiencies in *NPC1^-/-^*mice:  The locomotor function and motor coordination of *NPC1^-/-^* mice mice, estimated by the rota-rod test, were significantly improved by the treatment with 17b-estradiol. Tremors were first detected at 62.3 ± 0.4 days in 17b-estradiol-treated mice, significantly delayed as compared with 51.2 ± 0.5 days in untreated mice (P < 0.01, n = 15). There was no difference in survival, locomotor function, or motor coordination between male and female mice receiving 17b-estradiol treatment (data not shown). Thus, 17b-estradiol treatment significantly improved behavioral deficiencies in *NPC1^-/-^* mice. |
| Griffin et al. 2004 [6] | BALB/c NP-C mouse | Mice received one of three treatments: 0.5–2 μM ALLO in drinking water, with the concentration increasing by 0.5 μM every 2 weeks; a subcutaneous time-release pellet (250 mg over 90 d); or a single subcutaneous injection of 25 mg/kg of ALLO in 20%  HPßCD (2-hydroxypropyl-β-cyclodextrin), corresponding to 1.25 mg of ALLO per ml of 20% HPßCD. | Behavioral tests assessed locomotion, balance and coordination.  The open-field test graded locomotion and exploratory movements, incorporating an analysis of paw placement and position, limb movement, trunk position, stability and coordination of stepping, into the 21-point score.  Strength and motor coordination (10 points) were evaluated by the maximal angle of an inclined plane that the mouse could scale and the ‘tightrope test’, which assesses the mouse’s ability to stay on a 12-inch rope suspended between two poles (hang time, wrapping its tail, placing all four paws on the rope and traveling the length of the rope). | Adding ALLO to the drinking water of weaned *NPC1* mice at P21–P23 increased the lifespan of *NPC1* mice from 67 to 80 d (*n* = 12 mice in each group; *P* < 0.05), and implanting 90-d release pellets containing 250 g of ALLO under the skin of *NPC1* mice at P21–P23 (*n* = 12 mice) had a similar effect (mean survival 82 d; *P* < 0.01 versus *NPC1* mice receiving placebo pellets. Locomotor function and motor coordination declined at 8 weeks in both untreated and ALLO-treated *NPC1* mice, but the rate of decline was less in ALLO-treated *NPC1* mice. There was no difference in survival, locomotor function or motor coordination between male and female mice receiving ALLO treatment.  Earlier administration of ALLO gave better results. A single injection of ALLO (25 mg per kg body weight) at P23, P17, P10 or P7 progressively increased survival and delayed loss of locomotion, coordination and weight. Mice treated at P7 had a slight loss in motor coordination at 11 weeks and normal locomotion for 12 weeks. Mice treated at P10 also showed significant delays in the onset of tremors and ataxia as compared with untreated *NPC1* mice (*P* < 0.05), but these delays were not as great as those seen in mice treated at P7. There was no difference in survival, locomotor function or motor coordination between male and female mice receiving single injections of ALLO. |
| Hallows et al. 2006 [7] | Heterozygous BALB/cNctr-*Npc1mIN*/J (*npc^+/-^*). *npc^+/-^* mice were crossed with *p35^+/-^*mice having a C57BL/6 background to obtain double heterozygous (*npc^+/-^ p35^+/-^)* mice in the mixed BALB/c/C57BL/6 background. Double-heterozygous mice were mated to obtain *wt*, *p35^-/-,^ npc^-/-^*, and *npc^-/-,^ p35^-/-^*genotypes in the mixed background, and all comparisons between these different genotypes were made using littermates. | The p35 null mice have been useful for examining the role of cdk5/p25 in other types of neurodegeneration. | Mice were weighed and motor ability was assessed twice weekly between 3 and 10 weeks of age. Mice were allowed to grasp the bottom of a coat hanger suspended 18 cm above a flat surface and allowed to remain on the hanger for up to 2 min (hanging time). Their behavior on the hanger was also noted. Hanging time was multiplied by behavior to obtain a numeric value for motor ability. Values were averaged within genotypes at each time point (*n =* 13 wild type, 14 *p35^-/-^*, 10 *NPC^-/-^,* and 13 *NPC^-/-^, p35^-/-^*). | To determine whether weight loss or motor defects were improved in *NPC^-/-^* mice lacking p35/p25, mice were weighed and tested their motor ability twice weekly between the ages of 3 and 10 weeks of age. Both wild type and *p35^-/-^* mice continued to gain weight over the course of testing, whereas *NPC^-/-^* and *NPC^-/-^, p35^-/-^* mice lost weight beginning at ~ 6 weeks of age.  Motor ability held fairly constant in wild type and *p35^-/-^* mice. *NPC^-/-^*mice demonstrated a dramatic decline in motor ability beginning at ~ 6 weeks of age, whereas *NPC^-/-^, p35^-/-^* mice demonstrated this decline at an earlier time of ~ 5 weeks. Overall, these findings show no improvement in weight loss or motor ability in *NPC^-/-^* mice lacking p35/p25. |
| Hovakimyan et al. 2013 [8] | BALB/cNctr-Npc1m1N/-J | The COMBI-treated animals were injected with HPßCD/ALLO (25 mg/kg ALLO dissolved in 40% HPßCD) weekly, starting at postnatal day 7 (P7). These mice were injected with MIGLU dissolved in 0.9% NaCl solution, 300 mg/kg, between postnatal days P10 and P23. Starting at P23 the mice were fed powdered chow with daily addition of MIGLU. For the sham-treatment the HPßCD/ALLO and MIGLU injections were substituted. | Locomotor activity was assessed in open field, elevated plus maze and accelerod tests. For assessment of spatial learning and memory the Morris water maze test was conducted. | The sham-treated mutant mice exhibited motor impairments in all performed tests.  In the water maze the sham-treated mutants exhibited impairment in remembering the location of the hidden platform.  CYCLO/ALLO/MIGLU treatment positively influenced motor dysfunction: total distance and number of visits significantly increased, and accelerod performance improved. The spatial learning, however, did not benefit from therapy.  Open field. When compared to the sham-treated control mice, the total distance and number of total visits were significantly lower in the sham-treated mutant mice (p<0.001). The combination-treated mutant mice group revealed an increase in both parameters. When compared to the sham-treated mutant mice, the combination-treated ones travelled longer distances (4615 ± 1290 cm vs. 3195 ± 1291 cm) in the whole open field and revealed a higher number of total visits (283 ± 112 vs. 219 ± 83, p=0.051).  The data suggest that combined CYCLO/ALLO/MIGLU treatment resulted in significant improvements in spontaneous locomotor abilities in *NPC1* mutant mice.  Accelerod test. The mice of all groups acquired the accelerod test quickly and reached a stable level of performance before testing. No differences in the learning ability of mice of different groups could be observed. At postnatal day 35 the sham-treated controls and *NPC1* mutants revealed no significant differences in maximum speed, both exhibiting 21 rounds per minute (p=0.912). The combination-treated mutant mice, however, demonstrated an increase in maximum speed, reaching 34 rounds per minute. At time point P60 the accelerod performance was significantly (p<0.001) disrupted in the sham-treated *NPC1* mutant group (10 ± 4 rounds per minute) when compared to the sham-treated control group (21 ± 6 rounds per minute). The combination-treated mutant mice, however, demonstrated a significantly better performance when compared to the sham-treated ones, reaching 24 ± 7 rounds per minute (p<0.001). |
| Hung et al. 2016 [9] | BALB/cJ Npc1^nih^ (Npc1^+/–^) | The mice were monitored daily, and from P24, weighed every 2–3 days, then daily on weekdays from P36 until the end of the experiment at P71. | Mice were subjected to motor coordination and balance tests at the age of 5, 7, and 9 weeks. The tests were performed separately on consecutively days at the respective age.  Tests: Rotarod Test, Open-field Test and DigiGait Analysis. | To assess the effect of *NPC1* haploinsufficiency on motor coordination and balance, *NPC1*^+/+^, *NPC1^+/–^*, and *NPC1^–/–^* mice were tested on an accelerating Rotarod at 5, 7, and 9 weeks of age. The data were analyzed as distance travelled on the accelerating Rotarod, which took into account the rotation speed and latency to fall. There was no significant difference in the distance travelled on the Rotarod by mice of all genotypes between training and testing days (p =0.66). There was a significant interaction of genotype with age (p <0.0001), with the main significant effect between *NPC1^+/+^* and *NPC1^–/–^* mice, but not *NPC1^+/+^* and *NPC1^+/–^* mice, over age. The distance travelled by *NPC1^–/–^* mice was significantly less than *NPC1^+/+^* mice, and markedly decreased progressively with age. The distance travelled by *NPC1^+/–^* mice on the Rotarod did not differ significantly from that of *NPC1^+/+^* mice. However, examining the change in distance travelled at 7 and 9 weeks of age compared with 5 weeks of age, *NPC1^+/+^* mice showed improved motor coordination and balance with maturity (to 9 weeks), while *NPC1^+/–^* mice failed to mature in these domains. Male *NPC1^+/–^* and *NPC1^–/–^* mice were significantly less able to maintain their balance and coordination on the Rotarod compared with female *NPC1^+/–^* and *NPC1^–/–^* mice, but there was no sex difference in the Rotarod performance of *NPC1^+/+^* mice.  Compared with *NPC1^+/+^* mice, the total ambulatory distance travelled in the entire arena by the *NPC1^+/–^* mice was not different, whereas that of *NPC1^–/–^* mice was significantly decreased by 737.6 ± 112.6 cm. There was a significant interaction of genotype with age (p= 0.01), with the main significant effect detected between *NPC1^+/+^* and *NPC1^–/–^* mice over age. By 9 weeks of age, the total ambulatory distance travelled by *NPC1^+/+^* mice increased significantly by 183.2 ± 93.5 cm compared with 5 weeks of age (p=0.05), whereas there was no significant change detected in *NPC1^+/–^* and *NPC1^–/–^* mice. The average velocity of *NPC1^+/–^* mice movement was significantly slower than *NPC1^+/+^* mice, and intermediate between *NPC1^+/+^* and *NPC1^–/–^* mice. *NPC1^–/–^* mice exhibited significantly decreased ambulatory count in the total arena and center ambulatory count IRRs compared with *NPC1^+/+^* mice, consistent with motor impairment. The ambulatory count in the total arena of the *NPC1^+/–^* mice did not differ significantly from that of the *NPC1^+/+^* mice. However, the ambulatory count IRR of *NPC1^+/–^* mice in the center of the arena was only about 60 % that of *NPC1^+/+^* mice, indicative of increased anxiety. Male mice entered the center of the arena at about 60 % of the incidence rate of female mice. |
| Li et al. 2005 [10] | Heterozygous *NPC1* mice with a BALB/c background were mated. | This study defines the functional, biochemical, and molecular events that ensue as nerve cell death occurs. In most studies, the animals were weaned at 3 weeks of age onto a basal rodent diet (No. 7001 Harlan Teklad, Madison WI) that contained 0.02% (wt/wt) cholesterol. In some studies, groups of mice were placed on the same diet that had been enriched with 1.0% cholesterol (wt/wt) from the time of weaning until the end of the experiments. | The accelerating rotarod apparatus (Rotamex 4/8, Columbus Instruments, Columbus, OH) was used to measure motor coordination. Each mouse was placed on the rotating rod at a starting speed of 4 rpm and this speed was gradually increased to 40 rpm by 10 minutes. Four trials were carried out each day with a rest period of 1 hour between the first 2 trials  and the last 2 trials. | 4 groups of 16 mice each containing equal numbers of male and female animals were evaluated by quantifying neuromuscular coordination utilizing the rotarod apparatus. There was no significant effect of cholesterol feeding in the *NPC1^+/+^* animals between 3 and 11 weeks of age. However, the *NPC1^-/-^* animals began to show poorer coordination at 5 weeks of age, and the duration that they remained on the rotating rod rapidly declined thereafter reaching nearly 0 minutes at 11 weeks. Importantly, there was no effect of cholesterol feeding. |
| Nicoli et al. 2016 [11] | Npc1 mutant (BALB/cNctr-Npc1m1N/J, Npc1^-/-^), control (Npc1^+/+^) and Npc1 heterozygous mice (*NPC1^+/-^* mice) | *NPC1* mutant (BALB/cNctr-Npc1m1N/J, *NPC1^-/-^*) mice upon weaning at 3 weeks of age began receiving 4000 mg/kg HPBCD weekly delivered by IP injection.  Bile Acid Supplementation. *NPC1^-/-^* mice, *NPC1^+/-^* mice, and *NPC1^+/+^* mice (n = 6 per group) were fed either normal chow (RM1 maintenance diet; SDS, London, UK) or normal chow supplemented with ursodeoxycholic acid (0.5%, w/w) mixed with powdered diet. Treatment started at weaning (3 weeks of age) and mice were sacrificed at 6 and 9 weeks of age. | Motor function and coordination were assessed by observational counting of the total rearing events over a period of 5 minutes (either without support or against the cage wall) every week in the open-field. | Rearing (a measure of motor coordination) increased in the *NPC1^-/-^* mice treated with UDCA compared to the untreated *NPC1^-/-^* mice. While a high degree of variability was seen for all groups, UDCA-treated *NPC1^-/-^* mice showed marked improvements, which was significant at 5 weeks of age (p value = 0.0055) and from 7 to 12 weeks (p value = 0.050, p value = 0.002, p value = 0.013, p value = 0.001, p value = 0.0007 and p value = 0.002 respectively). No differences were detected between the *NPC1*^+/+^ treated or untreated mice. *NPC1^-/-^* mice on UDCA also retained motor function longer than untreated *NPC1^-/-^* mice as demonstrated by their ability to rear at 11 weeks, an age at which all untreated *NPC1^-/-^* mice are incapable of rearing (p value = 0.0007) |
| Rabl et al. 2016 [12] | BALB/cNctr-Npc1^m1N^/-J homozygous mice were bred by pairing one heterozygous male with one heterozygous female. Only male animals were longitudinally tested at the age of 6 and 8 weeks. | Authors established the pasta gnawing test by measuring the biting noise of mice that eat a piece of spaghetti. Two parameters were evaluated, the biting speed and the biting peaks per biting episode. Three mouse models of Parkinson’s disease, amyotrophic lateral sclerosis and Niemann-Pick disease were tested in the pasta gnawing test, RotaRod and wire suspension test. | The pasta gnawing test, RotaRod and wire suspension test. | Our results show that the pasta gnawing test reliably displays orofacial motor deficits. *NPC1^-/-^* mice showed first motor impairments in the pasta gnawing test at the age of 8 weeks compared to wildtype animals. Significant differences could be observed in the number of biting peaks per episode but not in the biting speed. Similar results were obtained using the Rota Rod test. The wire suspension time of *NPC1^-/-^* was not affected. The onset of motor deficits in *NPC1^-/-^* mice is thus comparable with already published results (Voikar et al., 2002; Zhang et al., 2004). |
| Santiago-Mujica et al. 2019 [13] | BALB/cNctr-Npc1<m1N>/J | No treatment.  *NPC1^–/–^* mice were characterized for their hepatic and neuronal phenotype to confirm the stability of the phenotype, provide a characterization of disease progression and pinpoint the age of robust phenotype onset. Animals of 4-10 weeks of age were analysed for general health, motor deficits as well as hepatic and neuronal alterations with a special focus on cerebellar pathology. | To assess motor coordination, the Rota Rod and Beam Walk test were performed. | *NPC1^-/-^* mice showed a progressive decrease in the latency to fall off of the rod. When compared to wild type littermates, significant differences were observed at 8-10 weeks of age.  The Beam Walk test was performed only with 7 week old animals since older animals coordination between *NPC1^-/-^* mice and wild type littermates could be observed at each of the five beams used. *NPC1^-/-^* mice presented a significantly increased number of slips compared to wild type littermates. The highest amount of slips was observed on the square beam with 10 mm diameter, but even on the 28 mm round beam animals performed significantly worse than wild type littermates. |
| Schlegel et al. 2016 [14] | 81 male wild type mice BALB/c-npc1nihNPC1 | Starting at postnatal day 7 (P7) and thenceforth, mice of the COMBI-group were injected weekly with HPßCD/ALLO (25 mg/kg ALLO dissolved in 40% HPßCD in Ringer’s solution, 4000 mg/kg, i.p.). Additionally, these mice were daily injected with MIGLU, dissolved in 0.9% NaCl solution, 300 mg/kg i.p. from P10 to P23. From P23 onwards until termination of experiments mice were fed standard chow with embedded MIGLU resulting in daily intake of 1200 mg/kg MIGLU. The MIGLU-group was treated like the COMBI-group, but without administration of HPßCD/ALLO, instead mice got vehicle. Mice of the sham-group were injected like those of the COMBI-group at the various time points with the respective volumes of 0.9% NaCl or without volume and were fed with chaw without drugs. | A battery of behavioral tests consisted of accelerod, Morris water maze, elevated plus maze, open field and hot plate tests was used | MIGLU-treated wild type mice displayed impaired spatial learning compared to sham- and COMBI-treated mice. Both COMBI- and MIGLU-treated mice showed enhanced anxiety in the elevated plus maze compared to sham-treated mice.  For evaluating motor coordination and balance, the accelerod test was performed. Animals of all groups learned the task during both training trials, indicated by decreasing numbers of down falls during the course of the training (P35: F14, 546 = 2.944, p < 0.001; P60: F14, 546 = 2.223, p = 0.006). Sham-treated mice started worse than MIGLU- and COMBI-treated mice. All pairwise multiple comparison procedures (Holm–Sidak method) revealed significant differences (p < 0.001) for training trials 1 and 2 at p35 and for training trial 1 at P60. However, during further training we did not detect any significant differences in motor performance between the three treatment groups. During probe trials with accelerating speed of the treadmill we also detected no statistically significant difference between the treatment groups with regard to reached speed at down fall at P35 and P60 (P35: F2, 79 = 2.012, p = 0.141; P60: F2, 79 = 2.684, p = 0.074). In conclusion, neither MIGLU nor COMBI treatment caused alteration of motor coordination and balance in comparison with sham-treated mice.  In the open field test, there was no difference in walking speed between all three groups (H2 = 5.605, p = 0.061). By analysing the ratio of center distance to total distance no significant differences between the three treatment groups (F2, 66 = 2.99, p = 0.057) were observed. In addition, significant differences between all three groups by analysis of the ratio of center to total visits were observed. |
| Võikar et al. 2002 [15] | Heterozygous Balb/C mice carrying the mutation in the NPC1 protein | The growth rate followed and behavioural methods applied in order to establish the onset and development of the major symptoms in the NPC mouse model. | Mice were subjected to a battery of coordination tests at the age of 28, 35, 42, 49 and 56 days. The battery consisted of four consecutive tests (vertical screen, beam balancing, coat hanger, rota-rod) in one session and each mouse carried out two sessions on the same day at the respective age. 1. Vertical screen (VS). 2. Balancing on a round beam (BB). 3. Coat hanger (CH). 4. Rota-rod (RR). 5. Spontaneous locomotor activity. At the age of 42 days the locomotor activity of the mice was assessed.  Videoanalyser registered the distance travelled by the animals in the open field arena (30×30×15 cm arena with transparent walls under reduced illumination, ~ 100 l× x) every 3 min during 15 min. 6. Learning and memory in Morris water maze. | The battery of coordination tests (vertical screen, beam balancing, coat hanger and rotating rod) established motor impairment of the *NPC1* mice already at the age of 28–42 days, well before the onset of visually detectable ataxia. Decreased exploratory activity and lack of habituation was revealed in the *NPC1* mice by open field test. The diseased mice were unable to learn and remember the location of the hidden escape platform in spatial water maze task suggesting cognitive impairment. In several tests the male *NPC1* mice were more affected than the females.  Rota-rod: The ability to walk on the rotating rod decreased very rapidly in the diseased mice during the observation period and again, the *NPC1* males were more affected [genotype *F*(1,50)=188.8, *P<*0.01; sex *F*(1,50)=5.4, *P*_0.05; age *F*(4,200)=53.7, *P<*0.01; genotype×sex *F*(1,50)=6.2, *P<*0.05]. The difference between the wild type and *NPC1* mice was first observed at 35 days in the male and at 42 days in the female group. |
| Williams et al. 2014 [16] | BALBc/NPC^nih^ | Ibuprofen (Sigma, 100 mg/kg/day) was supplemented as a dry admixture to powdered RM1 mouse chow (from 6 weeks of age). MIGLU (600 mg/kg/day) and curcumin (150 mg/kg/day) were administered as dry admixtures from 3 weeks of age. The untreated mice were fed on powdered chow. Treatment groups were made up of approximately equal numbers of males and females and received ibuprofen, curcumin, MIGLU, curcumin and ibuprofen, curcumin and MIGLU, or all three therapies. | Weight and spontaneous activity of each mouse were recorded weekly until reaching the late humane end-point (loss of 1 g body weight within 24 h). After 5–30 min room acclimatization, the mouse was placed in the ‘open field’ (measuring 45 × 25 × 12 cm). Rearing was recorded manually for 5 min (the number of times the mouse reared on its hind legs with or without support of the cage wall). | At 8 weeks of age, rearing was not significantly improved by ibuprofen or curcumin monotherapies but was improved by MIGLU monotherapy and any combination that included MIGLU (untreated *NPC1^−/−^*, 2.28 events/5 min compared to MIGLU, 22.64; curcumin & MIGLU, 28.82; and triple combination, 41.00 events/5 min, all p<0.001). Rearing activity was significantly improved in both the curcumin and MIGLU dual therapy (p< 0.01) and the triple combination therapy (p< 0.001) compared to MIGLU alone. At 11 weeks of age, only the dual combination of MIGLU and curcumin and the triple combination therapy maintained rearing over the untreated *NPC1^−/−^* group (p< 0.01 and p< 0.001, respectively). Strikingly, at this time point the triple combination group demonstrated significant maintenance of rearing activity when compared to the dual therapy (p <0.05). |

**References**

1. Ahmad, I.; Lope-Piedrafita, S.; Bi, X.; Hicks, C.; Yao, Y.; Yu, C.; Chaitkin, E.; Howison, C. M.; Weberg, L.; Trouard, T. P.; Erickson, R. P. Allopregnanolone treatment, both as a single injection or repetitively, delays demyelination and enhances survival of Niemann-Pick C mice. *J. Neurosci. Res.* **2005**, *82*, 811–821.

2. Alvarez, A. R.; Klein, A.; Castro, J.; Cancino, G. I.; Amigo, J.; Mosqueira, M.; Vargas, L. M.; Yévenes, L. F.; Bronfman, F. C.; Zanlungo, S. Imatinib therapy blocks cerebellar apoptosis and improves neurological symptoms in a mouse model of Niemann-Pick type C disease. *FASEB J.* **2008**, *22*, 3617–3627.

3. Bascuñan-Castillo, E. C.; Erickson, R. P.; Howison, C. M.; Hunter, R. J.; Heidenreich, R. H.; Hicks, C.; Trouard, T. P.; Gillies, R. J. Tamoxifen and vitamin E treatments delay symptoms in the mouse model of Niemann-Pick C. *J. Appl. Genet.* **2004**, *45*, 461–467.

4. Borbon, I. A.; Hillman, Z.; Duran, E.; Kiela, P. R.; Frautschy, S. A.; Erickson, R. P. Lack of efficacy of curcumin on neurodegeneration in the mouse model of Niemann-Pick C1. *Pharmacol. Biochem. Behav.* **2012**, *101*, 125–131.

5. Chen, G.; Li, H. M.; Chen, Y. R.; Gu, X. S.; Duan, S. Decreased estradiol release from astrocytes contributes to the neurodegeneration in a mouse model of Niemann-Pick disease type C. *Glia* **2007**, *55*, 1509–1518.

6. Griffin, L. D.; Gong, W.; Verot, L.; Mellon, S. H. Niemann-Pick type C disease involves disrupted neurosteroidogenesis and responds to allopregnanolone. *Nat. Med.* **2004**, *10*, 704–711.

7. Hallows, J. L.; Iosif, R. E.; Biasell, R. D.; Vincent, I. p35/p25 is not essential for tau and cytoskeletal pathology or neuronal loss in Niemann-Pick type C disease. *J. Neurosci.* **2006**, *26*, 2738–2744.

8. Hovakimyan, M.; Maass, F.; Petersen, J.; Holzmann, C.; Witt, M.; Lukas, J.; Frech, M. J. J.; Hübner, R.; Rolfs, A.; Wree, A. Combined therapy with cyclodextrin/allopregnanolone and miglustat improves motor but not cognitive functions in Niemann-Pick Type C1 mice. *Neuroscience* **2013**, *252*, 201–11.

9. Hung, Y. H.; Walterfang, M.; Churilov, L.; Bray, L.; Jacobson, L. H.; Barnham, K. J.; Jones, N. C.; O’Brien, T. J.; Velakoulis, D.; Bush, A. I. Neurological Dysfunction in Early Maturity of a Model for Niemann–Pick C1 Carrier Status. *Neurotherapeutics* **2016**, *13*, 614–622.

10. Li, H.; Repa, J. J.; Valasek, M. A.; Beltroy, E. P.; Turley, S. D.; German, D. C.; Dietschy, J. M. Molecular, anatomical, and biochemical events associated with neurodegeneration in mice with Niemann-Pick type C disease. *J. Neuropathol. Exp. Neurol.* **2005**, *64*, 323–333.

11. Nicoli, E. R.; Eisa, N. Al; Cluzeau, C. V. M.; Wassif, C. A.; Gray, J.; Burkert, K. R.; Smith, D. A.; Morris, L.; Cologna, S. M.; Peer, C. J.; Sissung, T. M.; Uscatu, C. D.; Figg, W. D.; Pavan, W. J.; Vite, C. H.; Porter, F. D.; Platt, F. M. Defective cytochrome p450-catalysed drug metabolism in Niemann-Pick type C disease. *PLoS One* **2016**, *11*.

12. Rabl, R.; Horvath, A.; Breitschaedel, C.; Flunkert, S.; Roemer, H.; Hutter-Paier, B. Quantitative evaluation of orofacial motor function in mice: The pasta gnawing test, a voluntary and stress-free behavior test. *J. Neurosci. Methods* **2016**, *274*, 125–130.

13. Santiago-Mujica, E.; Flunkert, S.; Rabl, R.; Neddens, J.; Loeffler, T.; Hutter-Paier, B. Hepatic and neuronal phenotype of NPC1 −/− mice. *Heliyon* **2019**, *5*.

14. Schlegel, V.; Thieme, M.; Holzmann, C.; Witt, M.; Grittner, U.; Rolfs, A.; Wree, A. Pharmacologic treatment assigned for Niemann Pick Type C1 disease partly changes behavioral traits in wild-type mice. *Int. J. Mol. Sci.* **2016**, *17*, 1866.

15. Võikar, V.; Rauvala, H.; Ikonen, E. Cognitive deficit and development of motor impairment in a mouse model of Niemann-Pick type C disease. *Behav. Brain Res.* **2002**, *132*, 1–10.

16. Williams, I. M.; Wallom, K. L.; Smith, D. A.; Al Eisa, N.; Smith, C.; Platt, F. M. Improved neuroprotection using miglustat, curcumin and ibuprofen as a triple combination therapy in Niemann-Pick disease type C1 mice. *Neurobiol. Dis.* **2014**, *67*, 9–17.
