# Supplementary material for: Gender-Specific Effects of Two Treatment Strategies in a Mouse Model of Niemann-Pick Disease Type C1
Source: Int J Mol Sci. 2021 Mar 3;22(5):2539. doi: 10.3390/ijms22052539 (PMC7962008; doi:10.3390/ijms22052539)
Supplement: Supplementary file 1 [file ijms-22-02539-s001.zip › Supplement Figure 1.docx]

|  | male | COMBI | | MIGLU | | HPßCD | | HPßCD1x | |
| --- | --- | --- | --- | --- | --- | --- | --- | --- | --- |
| female |  |  |  |  |  |  |  |  |  |
| body weight | |  | ↓ |  | ─ |  | ─ |  | ─ |
|  |  | − |  | − |  | − |  | − |  |
| brain weight | |  | ↓ |  | ─ |  | ─ |  | ─ |
|  |  | ↓ |  | − |  | − |  | − |  |
| brain/body weight | |  | ─ |  | ─ |  | ─ |  | ─ |
|  |  | − |  | − |  | − |  | − |  |
| anesthetic consumption | |  | ─ |  | ─ |  | ─ |  | ─ |
|  |  | − |  | − |  | − |  | − |  |
| anesthetic/body weight | |  | ─ |  | ─ |  | ─ |  | ─ |
|  |  | − |  | − |  | − |  | − |  |
| anesthetic/brain weight | |  | ─ |  | ─ |  | ─ |  | ─ |
|  |  | − |  | − |  | − |  | − |  |
| accelerod test | |  | ─ |  | ─ |  | ─ |  | ─ |
|  |  | − |  | − |  | − |  | − |  |
| OF test,  total distance | |  | ─ |  | ─ |  | ─ |  | ─ |
|  |  | − |  | − |  | − |  | − |  |
| OF test, relative center distance | |  | ─ |  | ─ |  | ─ |  | ─ |
|  |  | − |  | − |  | − |  | − |  |

**Supplementary Figure 1** Changes, induced by COMBI, MIGLU, HPßCD and HPßCD1x in *NPC1^+/+^* mice. Deterioration (↓) or no significant change (−) compared to the respective None- and/or Sham-groups.
