# Supplementary material for: Gender-Specific Effects of Two Treatment Strategies in a Mouse Model of Niemann-Pick Disease Type C1
Source: Int J Mol Sci. 2021 Mar 3;22(5):2539. doi: 10.3390/ijms22052539 (PMC7962008; doi:10.3390/ijms22052539)
Supplement: Supplementary file 1 [file ijms-22-02539-s001.zip › Supplement-Table 1.docx]

**Supplementary Table 1**. Overview of body and brain weights of *NPC1* mice treated with various drugs.

| **Citation** | **Mice Strain, gender** | **Treatment** | **Body weight** | **Brain weight** |
| --- | --- | --- | --- | --- |
| Ahmad et al. 2005 [1] | *Npc1*^NIH^ mutant (*NPC1^-/-^*) mice on the BALB/cJ background or a mixed BALB/cJ x FVB background with the mdr1a knockout. | ALLO (Sigma, St. Louis, MO) was dissolved in 20% HPßCD in water, at 1.25 mg/ml, and injected at 25 mg/kg subcutaneously at day 7, and intraperitoneally at later times. In the case of repeated injections, only the *NPC1^-/-^* mice were again injected on day 21 and at 2-week intervals thereafter until death. | Weight loss was significantly delayed in mice treated with ALLO on day 7. Treated double-mutant *NPC1^-/-^mdr1a^-/-^* mice were delayed in their attainment of the usual maximal weight, but then declined in parallel with the *NPC1^-/-^*non-*mdr1a^-/-^* mice. | Not done. |
| Alvarez et al. 2008 [2] | BALB/c mice carrying a heterozygous mutation in the *npc1*gene | Mice received daily intraperitoneal injections of imatinib mesylate (Gleevec; Novartis, Basel, Switzerland) (5 mg/kg in 0.9% NaCl) from postnatal day (p) 7 or 28. Control groups (wild type and *NPC1* mice) received daily intraperitoneal injections of 0.9% NaCl. | Imatinib delayed loss of weight in the *NPC1* mice. Weight started to decline at 7 wk in both untreated and imatinib-treated *NPC1* mice from p7 and p28, but the rate of decline was reduced in imatinib-treated *NPC1* mice. Earlier administration of imatinib seemed to give better results, but there was no significant difference between the gain of weight curves of *NPC1* mice treated from p7 or p28. There was no difference in the weight gain between imatinib-treated and untreated wild type mice. | Not done. |
| Bascunan-Castill et al. 2004 [3] | *Npc1*^NIH^ mutant mice from the BALB/cJ background | Two drugs were used as a treatment in disease progression. Tamoxifen (desiccate, 99% concentrate, from Sigma-Aldrich, St. Louis, MO) was prepared by dissolving 16 µg of the drug per 1 ml of peanut oil at room temperature. Peanut oil used as a control. D-alpha tocopherol (brand “Natural Liquid Vitamin E”), was purchased from Solgar.  Treatment was initiated at day 21 mice receiving tamoxifen were injected intraperitoneally with the drug suspended in peanut oil on a weekly basis with a dosage delivering 0.023 µg/g/day. Mice receiving vitamin E were given 70 µl (25 IU) of D-alpha tocopherol through oral. | Tamoxifen treatment delayed weight loss in male and female *Npc1*^NIH^ mice compared to untreated controls, and maintained Rota-Rod performance for about an extra week. Vitamin E treatment delayed weight loss in females only. Simultaneous use of both treatments did not delay weight loss longer than tamoxifen-only treatment but had a greater effect than either treatment alone on Rota-Rod performance. | Not done. |
| Beltroy et al. 2005 [4] | Wild type (*NPC1^+/+^)* and homozygous mutant (*NPC1^-/-^*) mice were generated from heterozygous  *NPC1^+/-^* animals with a BALB/c background. | The studies delineate the biochemical, histological, and molecular abnormalities that occur in the liver of the NPC mouse maintained on low levels of dietary lipid intake.  All animals were fed *ad libitum* a low-cholesterol rodent diet after weaning at 19 days of age. This diet had a cholesterol content of 0.016% (wt/wt) and a total lipid content of 5% (wt/wt). Measurements were made in animals at different ages, varying from 1 to 75 days. | The growth of the whole animal was quantified, and the absolute and relative weights of the liver and brain. The *NPC1^+/+^* and *NPC1^-/-^* animals progressively gained weight over most of the 75 days, although a pause occurred in the rate of gain in both groups just as the animals were weaned to solid food.  The mean weights of the *NPC1^-/-^* animals were lower than those of the *NPC1^+/+^* mice at nearly every age, and these differences became large between 49 and 75 days of age. | The absolute weight of the brain was less in mutant mice. However, when corrected for the lower body weights seen in these  *NPC1^-/-^* animals, relative brain weight was the same in the mutant and control mice. |
| Caporali et al. 2016 [5] | Npc1nmf164/nmf164 mice with BALB/cJ background (hereafter named Npc1nmf164 mice) obtained from heterozygous crosses. | Because sensorimotor reflexes and motor skills normally appear with a definite timing during the first 3 weeks after birth, they represent a useful tool to assess early postnatal neural development. Acquisition of several developmental milestones in the physical and sensorimotor development of Npc1nmf164 mice from PN3 until weaning (PN21) were evaluated. | Body weight, fur appearance, incisor eruption and eye opening were recorded as indexes of physical growth and development, observing no difference between Npc1nmf164 and wild type littermates. All pups similarly increased their body weight in the interval PN3-PN21, showed dorsal and ventral fur appearance after PN5, incisor eruption after PN7, and eye opening after PN14. | Not done. |
| Chandler et al. 2017 [6] | BALB/cNctr-Npc1m1N/J strain | To test the efficacy of gene therapy as a treatment for NPC1 at different time points, *NPC1^-/-^* mice received retro-orbital injections of AAV9-CamKII-NPC1 during the neonatal period (n=6; dose 2.6x10^11^ GC), or at weaning on DOL 20–25 (n=9; dose 1.3x10^12^ GC). Another group of *NPC1^-/-^* mice (n=6; dose 1.2x10^12^ GC) received AAV9-CamKII-GFP by retro-orbital injection at DOL 20–25 and served as treatment controls. | Gene delivery had no significant effect on mean weight between 4 and 9 weeks. While both untreated (n=16) and AAV9-CamKII-GFP-treated *NPC1^-/-^* mice (n=6) almost uniformly reach their peak weights at 6 weeks, *NPC1^-/-^* pups (n=6) or weanlings (n=9) that received AAV9-CamKII-NPC1 reached peak weights at 8 weeks (P<0.01 and P<0.1, respectively). Percent weight change from 6 to 9 weeks was also compared between untreated, AAV9-CamKII-NPC1 and AAV9-CamKII-GFP treated *NPC1^-/-^* mice because the weight decline of untreated mice began at 6 weeks, and most untreated mice did not survive beyond 9 weeks. Untreated and AAV9-CamKII-GFP-treated *NPC1^-/-^* mice had a percent weight change of -17% and -23%, respectively, between 6 and 9 weeks. Relative to untreated *NPC1^–/–^* mice, AAV9-CamKII-NPC1 *NPC1^-/-^* pups treated as neonates or at weaning demonstrated significantly less weight loss [-3.8% (P<0.02) and -2.3% (P<0.001), respectively] over this same time period. | Not done. |
| Chen et al. 2007 [7] | Mice were bred to produce normal (*NPC1^+/+^*), heterozygous (*NPC^+/-^*), and homozygous affected (*NPC1^-/-^*) mice. | Estradiol Treatment: *NPC1^-/-^* mice received subcutaneous injections of 2 mg/kg or 20 mg/kg of 17b-estradiol in corn oil weekly from P7; the concentrations of 17b-estradiol were 10 ng/ 10 lL or 100 ng/10 lL. | The untreated *NPC1^-/-^* mice had their maximal body weight approximately at 40 days (15.7 ± 2.1 g) and begin to loss their weight quickly afterward. Two doses of treatment with 17b-estradiol delayed the time starting to lose weight (50 days in treated mice vs. 45 days in untreated group) and attenuated the severity of weight loss estimated during 45–55 days. | Not done. |
| Davidson et al. 2009 [8] | *NPC1* mouse  (BALBc/NPC^nih^), males and females | Starting at P7 and weekly thereafter, some mice were  injected SC with either 20% HPßCD (control; 4000 mg/kg; H107, Sigma Aldrich, St. Louis, MO) or ALLO (dissolved in 20% HPßCD; 25 mg/kg; P3800-000, Steraloids, Newport, RI).  Additionally, some mice were injected IP every day starting at P10 until P23 with either saline (control; 0.9% normal saline; 104 6816, Fisher Scientific, Waltham, MA) or MIGLU (dissolved in saline; 300 mg/kg).  *NPC1^-/-^* mice treated with a combination therapy (COMBI) of MIGLU (administered daily starting at P10) and ALLO/HPßCD (administered weekly starting at P7).  Starting at P7 and weekly thereafter, some mice were injected SC with 20% HPßCD (control; 4000 mg/kg; H107, Sigma Aldrich, St. Louis, MO).  Chronic HPßCD injections: (injections given every other day), *NPC1^-/-^* mice and wild type mice were administered HPßCD beginning at P7 and continuing to end-stage disease. | COMBI treatment: Untreated *NPC1^-/-^* mice began a precipitous weight loss beginning at 6 weeks of age, while combination-treated *NPC1^-/-^* mice gradually lost weight starting at 14 weeks.  Weekly HPßCD injection: Weight loss in HPßCD-injected *NPC1^-/-^* mice was delayed by approximately 3 weeks.  Chronic HPßCD treatment: Abrupt weight loss in untreated *NPC1^-/-^* mice began to occur around 7 weeks of age, while treated *NPC1^-/-^* mice exhibited a gradual decline in weight beginning at 10 to 13 weeks of age. | Not done. |
| Griffin et al. 2004 [9] | BALB/c NPC mouse | Mice received one of three treatments: 0.5–2 μM ALLO in drinking water, with the concentration increasing by 0.5 μM every 2 weeks; a subcutaneous time-release pellet (250 mg over 90 d); or a single subcutaneous injection of 25 mg/kg of ALLO in 20%  HPßCD (2-hydroxypropyl-β-cyclodextrin; Sigma), corresponding to 1.25 mg of ALLO per ml of 20% HPßCD. | Earlier administration of ALLO gave better results. A single injection of ALLO (25 mg per kg body weight) at P23, P17, P10 or P7 progressively increased survival and delayed loss of locomotion, coordination and weight. | Not done. |
| Hallows et al. 2006 [10] | Heterozygous BALB/cNctr-*Npc1mIN*/J (*NPC1^–/–^)* were crossed with *p35^–/–^*mice having a C57BL/6 background to obtain double heterozygous (*NPC1^–/–^ p35^–/–^*) mice. | Our p35 null mice have been useful for examining the role of cdk5/p25 in other types of neurodegeneration. | To determine whether weight loss or motor defects were improved in *NPC1^–/–^* mice lacking p35/p25, mice were weighed and  tested their motor ability twice weekly between the ages of 3 and 10 weeks of age. Both wild type and *p35^–/–^* mice continued to gain weight over the course of testing, whereas *NPC1^–/–^* and *NPC1^–/–^p35^–/–^* mice lost weight beginning appr. 6 weeks of age. | Not done. |
| Hovakimyan et al. 2013 [11] | BALB/cNctr-Npc1m1N/-J | The COMBI-treated animals were injected with HPßCD/ALLO (25 mg/kg ALLO dissolved in 40% HPßCD, Sigma, Seelze, Germany) weekly, starting at postnatal day 7 (P7). These mice were injected with MIGLU dissolved in 0.9% NaCl solution, 300 mg/kg, between postnatal days P10 and P23. Starting at P23 the mice were fed powdered chow with daily addition of MIGLU. | There were no changes in body weight between all three groups for up to P45. At that time point the body weight was 11 ± 1.5 and 9 ± 1.3 g for sham-treated control and mutant mice, respectively. The combination-treated mutants revealed 10 ± 1.2 g, which was not different from that in sham-treated ones. On P65, the body weight of sham-treated mutant mice was significantly reduced when compared to the sham-treated controls (12 ± 1.1 vs. 17 ± 1.8 g; p<0.01). The combination-treated mutant mice, however, revealed a body weight of 16±1.6 g, which was significantly higher when compared to the weight of sham-treated mutants (p<0.01). | Not done. |
| Hung et al. 2016 [12] | BALB/cJ Npc1^nih^ (*Npc1*^+/–^) | Heterozygotes of a natural loss-of-function mutant NPC1 mouse model were examined.  The mice were monitored daily, and from P24, weighed every 2–3 days, then daily on weekdays from P36 until the end of the experiment at P71. | Fed a standard diet, there was no body weight difference between mice of all genotypes up to P28. We observed from P29 onwards that male mice became significantly heavier. Over the course of the present study (71 days), the age dependent gain in body weight was similar between sex matched *NPC1^+/+^* and *NPC1^+/–^* littermates. Compared with *NPC1^+/+^* and *NPC1^+/–^* littermates, the body weight of *NPC1^-/-^* mice decreased dramatically from 7 weeks of age for males and from 8 weeks of age for females. | Not done. |
| Jelinek et al. 2010 [13] | BALB/cJ *Npc1^NIH^* | The NPC1 heterozygous mouse model (*NPC1^+/−^*), was used to determine whether decreased *Npc1* gene dosage was associated with weight gain when fed either a low-fat (10% kcal fat) or high-fat (45% kcal fat) diet beginning at 4 weeks of age until 20 weeks of age. | *NPC1*^+/−^ mice had significantly increased weight gain beginning at 13 weeks of age when fed a high-fat diet, but not when fed a low-fat diet, compared to the *NPC1*^+/+^ mice fed the same diet. With respect to mice fed a high-fat diet, the *NPC1*^+/−^ mice continued to have significantly increased weight gain to 30 weeks of age. At this age, the *NPC1*^+/−^ mice were found to have increased liver and inguinal adipose weights compared to the *NPC1*^+/+^ mice. Decreased *Npc1* gene dosage resulting in decreased Npc1 protein function, promoted weight gain in mice fed a high-fat diet consistent with a gene–diet interaction. | Not done. |
| Jelinek et al. 2012 [14] | BALB/cJ Npc1^nih^ heterozygous (*NPC1^+/−^*) mice were bred to generate *NPC1* wild type (*NPC1^+/+^*) mice and *NPC1* heterozygous (*NPC1^+/−^*). | One week after weaning (28 days of age), the female *NPC1^+/+^* and *NPC1^+/−^* mice were weighed and placed on either a regular/low-fat or high-fat diet. The *NPC1^+/+^* and *NPC1^+/−^* mice were weighed every week from 5 to 25 weeks of age. At about 30 weeks of age, the *NPC1^+/+^* and *NPC1^+/−^* mice fed either the regular or the high-fat diet were killed and the liver weights and the ovarian fat pad weights were determined. | No significant differences in weight curves were found between female *NPC1^+/−^* and *NPC1^+/+^* mice when fed a regular diet. However, when placed on the high fat diet, female *NPC1^+/−^* were heavier than female *NPC1^+/+^*  mice from week 9 onwards and the difference had become significant from 18 weeks onwards. At 30 weeks of age, the *NPC1^+/−^* mice on the high fat diet had a significantly increased (23.3%) body weight compared to *NPC1^+/+^* mice fed the same high-fat diet. | Not done. |
| Kulkarni et al. 2018 [15] | Npc1^nmf164^ mice with BALB/cJ | To determine the efficacy of ORX-301 prodrug Npc1^nmf164^ and age matched wild type mice were subjected weekly to a subcutaneous injection of a 16% w/v ORX-301 solution in PBS (800 mg/kg body weight). Control group mice received plain PBS. Npc1^nmf164^ and wild type mice were randomly assigned to the various experimental groups. In the late intervention study, Npc1^nmf164^ and wild type mouse littermates were treated weekly with either ORX-301 or PBS starting from the 7th week of age. | The body weight was recorded weekly and a score ranging from 0 to 5 was assigned to each mouse. To analyze the effect of ORX-301 treatment on body weight loss, a score ranging from 0 to 5 assigned to each mouse, with higher score corresponding to higher severity of weight loss.  Sham-treated Npc1^nmf164^ mice obtained higher scores at weeks 12–14 when compared to those obtained by ORX-301-treated mice, suggesting that ORX-301 treatment slowed the progression of body weight loss. | Not done. |
| Li et al. 2005 [16] | Heterozygous *NPC1* mice with a BALB/c background were mated. | This study defines the functional, biochemical, and molecular events that ensue as nerve cell death occurs.  In most studies, the animals were weaned at 3 weeks of age onto a basal rodent diet (No. 7001 Harlan Teklad, Madison WI) that contained 0.02% (wt/wt) cholesterol. In some studies, groups of mice were placed on the same diet that had been enriched with 1.0% cholesterol (wt/wt) from the time of weaning until the end of the experiments. To first evaluate the interaction of gender and cholesterol intake, 4 groups of *NPC1^–/–^* and *NPC1^+/+^* mice were weaned onto diets containing either 0.02% or 1.0% cholesterol and observed for the duration of their lives. | In the *NPC1^+/+^* control animals, the gain in body weight was identical in the mice on the high cholesterol diet compared with those on the low cholesterol intake, although after 11 weeks both groups of male mice had reached body weights of approximately 25 g while both groups of the female animals achieved body weights that averaged only about 20 g. In contrast, at virtually every age beyond 4 weeks, the body weights in the *NPC1^–/–^* animals, male or female, were significantly less than those found in control animals. Feeding the high cholesterol diet did not reduce survival of either the male or female *NPC1^–/–^* animals. | Not done. |
| Liu et al. 2008 [17] | Mice were lacking functional NPC1 protein (*NPC1^–/–^*) on a BALB/c background. A second group of similar *NPC1^–/–^* mice was derived from heterozygous founders from the Jackson Laboratories (BALB/cNctr-Npc1^m1N^/J; stock number 003092). | All mice were fed ad libitum a low cholesterol (0.02%, w/w) rodent diet or the ground meal form of this same diet containing either cholesterol (1%, w/w) or an LXR agonist (T0901317). Intake of this latter diet provided each animal with an approximate daily dose of T0901317 equal to 50 mg/kg body weight. Other groups of mice were administered a single sc. injection at 7 days of age of a 20% (in saline) solution of HPßCD (4,000 mg/kg body weight). In addition, in some experiments, ALLO (5a-pregnan-3a-ol-20-one) was added to the HPßCD solutions at a concentration of 1.5 mg/ml (to provide 25 mg/kg body weight). | Body weight in 56-day-old *NPC1^+/+^* was significantly larger that in *NPC1^–/–^* mice. | Not done. |
| Lopez et al. 2014 [18] | Control (*NPC1^+/+^*) and homozygous (*NCP1^−/−^*) mice were generated from heterozygous (*NPC1^+/−^*) breeding stock on a pure BALB/c background. | *NPC1^−/−^* and *NPC1^+/+^* mice that had not received any prior treatments were administered weekly a subcutaneous injection at the scruff of the neck of either normal saline or a 20% (w/v) solution (in saline) of 2HPβCD (Sigma-Aldrich Corp; product H107) starting when they were 49-days old (approximate dose of 4000 mg/kg bw). Matching groups of 49-day old *NPC1^−/−^* and *NPC1^+/+^* mice received their respective treatments (saline vs 2HPβCD) at 49, 56, 63, and 70 days of age. At 77 days of age these mice were anesthetized, exsanguinated, and multiple organs were taken. | The average body weights of the baseline 49-day old *NPC1^+/+^* and *NPC1^−/−^* mice were 21.4 ± 1.3 and 19.7 ± 0.8 g, respectively. The trend toward a lower body weight in the *NPC1* mutants at 49 days of age became considerably more pronounced over the ensuing 28 days. | Not done. |
| Maass et al. 2015 [19] | BALB/c-npc1^nih^ | At P7 and thenceforth, *NPC1^–/–^* mice were injected weekly with ALLO (25 mg/kg) dissolved in HPßCD (4,000 mg/kg, i.p.). At P10 and until P23, animals were injected daily with MIGLU (300 mg/kg, i.p.). From P23 onward, animals were fed with MIGLU as powdered chow (1,200 mg/kg per day) until termination. | Not done | Brain weight of *NPC1^+/+^* sham mice was 0.475 ± 0.006 g. Brain weights were significantly decreased in *NPC1^–/–^* sham (0.389 ± 0.005 g) and *NPC1^–/–^* COMBI (0.387 ± 0.004 g). |
| Nicoli et al. 2016 [20] | *NPC1* mutant (BALB/cNctr-Npc1m1N/J, Npc1^-/-^), controls (Npc1^+/+^) and *NPC1^+/-^* were generated | *NPC1* mutant (BALB/cNctr-Npc1m1N/J, Npc1^-/-^) mice upon weaning at 3 weeks of age began receiving 4000mg/kg HPBCD weekly delivered by IP injection.  Bile Acid Supplementation. *NPC1*^-/-^, *NPC1*^+/-^ and *NPC1*^+/+^ mice were fed either normal chow or normal chow supplemented with ursodeoxycholic acid (0.5%, w/w) in powdered diet. Treatment started at 3 weeks of age and mice were sacrificed at 6 and 9 weeks of age. | Monitoring body weight revealed a similar pattern of weight gain and loss in *NPC1*^-/-^ mice regardless of treatment. However *NPC1^-/-^* mice treated with UDCA had a generalized reduction weight that was significant at 5, 6, 9, and 11 weeks compared with the untreated *NPC1*^-/-^ mice. This lower body weigh in the UDCA (ursodeoxycholic acid, UDCA) treated mice was also reflected in the *NPC1*^+/+^ mice with significant differences noted at 5, 11, and 12 weeks. No significant differences between genders were noted so genders were combined. | Not done. |
| Porter et al. 2010 [21] | BALB/c *NPC^nih^* mice. | Metabolites in the plasma and tissues of the *NPC1*^−/−^ mouse model were measured and several cholesterol oxidation products found that were elevated in *NPC1*^−/−^ mice, detectable prior to the onset of symptoms, and associated with disease progression. | In contrast to control wild type littermates, *NPC1*^−^*^/^*^−^ mice failed to appropriately gain weight and started to lose weight by 7–8 weeks of age. | Not done. |
| Santiago-Mujica et al. 2019 [22] | BALB/cNctr-Npc1<m1N>/J | No treatment.  Animals of 4-10 weeks of age were analysed for general health, motor deficits as well as hepatic and neuronal alterations with a special focus on cerebellar pathology. | *NPC1^–/–^* and wild type mice showed a similar body weight gain during the first 4 weeks of measurement was maintained more or less constant until mice were 8 weeks old. From 8 weeks onwards, *NPC1^–/–^* mice were significantly lighter compared to wild type littermates. | Not done. |
| Schlegel et al. 2016 [23] | 81 male BALB/c-npc1nihNPC1^-/-^ wild type mice (*NPC1^+/+^*) | Starting at postnatal day 7 (P7) and thenceforth, mice of the COMBI-group were injected weekly with HPßCD/ALLO (25 mg/kg ALLO dissolved in 40% HPßCD in Ringer’s solution, 4000 mg/kg, i.p.). Additionally, these mice were daily injected with MIGLU, 300 mg/kg i.p. from P10 to P23. From P23 onwards mice were fed standard chow with embedded MIGLU resulting in daily intake of 1200 mg/kg MIGLU. The MIGLU-group was treated like the COMBI-group, but without HPßCD/ALLO, instead mice got vehicle. Mice of the sham-group were injected like those of the COMBI-group with the respective volumes of 0.9% NaCl or without volume and were fed with chaw without drugs. | Mean body weight of sham-treated mice was 24.6 g (SD: 1.5 g), for COMBI-treated mice 22.5 g (SD: 1.6 g) and 24.4 g (SD: 1.3 g) for MIGLU-treated mice. Mean body weight of the COMBI-group was significantly reduced compared to the sham-group and the MIGLU-group, whereas body weights of sham- and MIGLU-groups did not significantly vary (p = 0.610). | Reduced brain weights after MIGLU- and COMBI treatment: Brains of combination- (mean: 0.408 g, SD: 0.0234 g) and MIGLU-treated mice (mean: 0.426 g, SD: 0.0156 g) were significantly lighter (p < 0.001) compared to sham-treated mice (mean 0.449 g, SD: 0.0278 g). |
| Võikar et al. 2002 [24] | Heterozygous Balb/C mice carrying the mutation in the NPC1 protein | The growth rate was followed and behavioural methods applied in order to establish the onset and development of the major symptoms in the NPC mouse model. | Both male and female *NPC1* mice displayed retarded growth at the age between 25 and 35 days. At the age of 35–45 days the weight was similar to controls and thereafter very rapidly decreased.  After day 45 the weight of *NPC1* mice started to decrease progressively. The weight loss was more dramatic in the male *NPC1* mice. By the age of 59 days the weight of the affected males was reduced to 85.7 ± 1.5% and that of affected females to 91.1 ± 1.6% of their reference value on day 45. | Not done. |
| Williams et al. 2014 [25] | BALBc/NPC^nih^ | Ibuprofen (Sigma, 100 mg/kg/day) was supplemented as a dry admixture to powdered RM1 mouse chow (SDS, UK) (from 6 weeks of age, due to the toxicity seen with earlier dosing, Smith et al., 2009). MIGLU (600 mg/kg/day, Oxford GlycoSciences/Celltech, UK) and curcumin (Sigma, 150 mg/kg/day) were administered as dry admixtures  as above (from 3 weeks of age). The untreated mice were fed on powdered chow (n = 34). Treatment groups were made up of approximately equal numbers of males and females and received ibuprofen (n = 14), curcumin (n = 11), MIGLU (n = 11), curcumin and ibuprofen (n = 5), curcumin and MIGLU (n = 11), or all three therapies (n = 9). | The weight of *NPC1^–/–^* mice plateaus at 7 weeks of age, and then progressively declines. While none of the treatment combinations significantly increased the maximal weight of *NPC1^–/–^* mice, significant alterations in the onset of weight loss were observed in MIGLU-treated mice and combinations containing MIGLU. These treatment regimens extended the length of time that *NPC1^–/–^* mice were able to gain and maintain weight, seen as a rightward shift of the typical untreated *NPC1^–/–^* weight curve. The mean weight of *NPC1^–/–^* mice was compared at 11 weeks of age when the average weight of the untreated *NPC1^–/–^* group had dropped to 13.16 g. While a number of monotherapies showed no significant maintenance of weight, the average weight of both the curcumin & MIGLU (16.1 g, p<0.05) and triple combination groups (18.1 g, p< 0.001) were significantly higher. | Not done. |

**References**

1. Ahmad, I.; Lope-Piedrafita, S.; Bi, X.; Hicks, C.; Yao, Y.; Yu, C.; Chaitkin, E.; Howison, C. M.; Weberg, L.; Trouard, T. P.; Erickson, R. P. Allopregnanolone treatment, both as a single injection or repetitively, delays demyelination and enhances survival of Niemann-Pick C mice. *J. Neurosci. Res.* **2005**, *82*, 811–821.

2. Alvarez, A. R.; Klein, A.; Castro, J.; Cancino, G. I.; Amigo, J.; Mosqueira, M.; Vargas, L. M.; Yévenes, L. F.; Bronfman, F. C.; Zanlungo, S. Imatinib therapy blocks cerebellar apoptosis and improves neurological symptoms in a mouse model of Niemann-Pick type C disease. *FASEB J.* **2008**, *22*, 3617–3627.

3. Bascuñan-Castillo, E. C.; Erickson, R. P.; Howison, C. M.; Hunter, R. J.; Heidenreich, R. H.; Hicks, C.; Trouard, T. P.; Gillies, R. J. Tamoxifen and vitamin E treatments delay symptoms in the mouse model of Niemann-Pick C. *J. Appl. Genet.* **2004**, *45*, 461–467.

4. Beltroy, E. P.; Richardson, J. A.; Horton, J. D.; Turley, S. D.; Dietschy, J. M. Cholesterol accumulation and liver cell death in mice with Niemann-Pick type C disease. *Hepatology* **2005**, *42*, 886–893.

5. Caporali, P.; Bruno, F.; Palladino, G.; Dragotto, J.; Petrosini, L.; Mangia, F.; Erickson, R. P.; Canterini, S.; Fiorenza, M. T. Developmental delay in motor skill acquisition in Niemann-Pick C1 mice reveals abnormal cerebellar morphogenesis. *Acta Neuropathol. Commun.* **2016**, *4*, 94.

6. Chandler, R. J.; Williams, I. M.; Gibson, A. L.; Davidson, C. D.; Incao, A. A.; Hubbard, B. T.; Porter, F. D.; Pavan, W. J.; Venditti, C. P. Systemic AAV9 gene therapy improves the lifespan of mice with Niemann-Pick disease, type C1. *Hum. Mol. Genet.* **2017**, *26*, 52–64.

7. Chen, G.; Li, H. M.; Chen, Y. R.; Gu, X. S.; Duan, S. Decreased estradiol release from astrocytes contributes to the neurodegeneration in a mouse model of Niemann-Pick disease type C. *Glia* **2007**, *55*, 1509–1518.

8. Davidson, C. D.; Ali, N. F.; Micsenyi, M. C.; Stephney, G.; Renault, S.; Dobrenis, K.; Ory, D. S.; Vanier, M. T.; Walkley, S. U. Chronic cyclodextrin treatment of murine Niemann-Pick C disease ameliorates neuronal cholesterol and glycosphingolipid storage and disease progression. *PLoS One* **2009**, *4*, e6951.

9. Griffin, L. D.; Gong, W.; Verot, L.; Mellon, S. H. Niemann-Pick type C disease involves disrupted neurosteroidogenesis and responds to allopregnanolone. *Nat. Med.* **2004**, *10*, 704–711.

10. Hallows, J. L.; Iosif, R. E.; Biasell, R. D.; Vincent, I. p35/p25 is not essential for tau and cytoskeletal pathology or neuronal loss in Niemann-Pick type C disease. *J. Neurosci.* **2006**, *26*, 2738–2744.

11. Hovakimyan, M.; Maass, F.; Petersen, J.; Holzmann, C.; Witt, M.; Lukas, J.; Frech, M. J. J.; Hübner, R.; Rolfs, A.; Wree, A. Combined therapy with cyclodextrin/allopregnanolone and miglustat improves motor but not cognitive functions in Niemann-Pick Type C1 mice. *Neuroscience* **2013**, *252*, 201–11.

12. Hung, Y. H.; Walterfang, M.; Churilov, L.; Bray, L.; Jacobson, L. H.; Barnham, K. J.; Jones, N. C.; O’Brien, T. J.; Velakoulis, D.; Bush, A. I. Neurological Dysfunction in Early Maturity of a Model for Niemann–Pick C1 Carrier Status. *Neurotherapeutics* **2016**, *13*, 614–622.

13. Jelinek, D.; Heidenreich, R. A.; Erickson, R. P.; Garver, W. S. Decreased Npc1 gene dosage in mice is associated with weight gain. *Obesity* **2010**, *18*, 1457–1459.

14. Jelinek, D. A.; Maghsoodi, B.; Borbon, I. A.; Hardwick, R. N.; Cherrington, N. J.; Erickson, R. P. Genetic variation in the mouse model of Niemann Pick C1 affects female, as well as male, adiposity, and hepatic bile transporters but has indeterminate effects on caveolae. *Gene* **2012**, *491*, 128–134.

15. Kulkarni, A.; Caporali, P.; Dolas, A.; Johny, S.; Goyal, S.; Dragotto, J.; Macone, A.; Jayaraman, R.; Fiorenza, M. T. Linear Cyclodextrin Polymer Prodrugs as Novel Therapeutics for Niemann-Pick Type C1 Disorder. *Sci. Rep.* **2018**, *8*.

16. Li, H.; Repa, J. J.; Valasek, M. A.; Beltroy, E. P.; Turley, S. D.; German, D. C.; Dietschy, J. M. Molecular, anatomical, and biochemical events associated with neurodegeneration in mice with Niemann-Pick type C disease. *J. Neuropathol. Exp. Neurol.* **2005**, *64*, 323–333.

17. Liu, B.; Li, H.; Repa, J. J.; Turley, S. D.; Dietschy, J. M. Genetic variations and treatments that affect the lifespan of the NPC1 mouse. *J. Lipid Res.* **2008**, *49*, 663–9.

18. Lopez, A. M.; Terpack, S. J.; Posey, K. S.; Liu, B.; Ramirez, C. M.; Turley, S. D. Systemic administration of 2-hydroxypropyl-β-cyclodextrin to symptomatic Npc1-deficient mice slows cholesterol sequestration in the major organs and improves liver function. *Clin. Exp. Pharmacol. Physiol.* **2014**, *41*, 780–7.

19. Maass, F.; Petersen, J.; Hovakimyan, M.; Schmitt, O.; Witt, M.; Hawlitschka, A.; Lukas, J.; Rolfs, A.; Wree, A. Reduced cerebellar neurodegeneration after combined therapy with cyclodextrin/allopregnanolone and miglustat in NPC1: A mouse model of Niemann-Pick type C1 disease. *J. Neurosci. Res.* **2015**, *93*, 433–442.

20. Nicoli, E. R.; Eisa, N. Al; Cluzeau, C. V. M.; Wassif, C. A.; Gray, J.; Burkert, K. R.; Smith, D. A.; Morris, L.; Cologna, S. M.; Peer, C. J.; Sissung, T. M.; Uscatu, C. D.; Figg, W. D.; Pavan, W. J.; Vite, C. H.; Porter, F. D.; Platt, F. M. Defective cytochrome p450-catalysed drug metabolism in Niemann-Pick type C disease. *PLoS One* **2016**, *11*.

21. Porter, F. D.; Scherrer, D. E.; Lanier, M. H.; Langmade, S. J.; Molugu, V.; Gale, S. E.; Olzeski, D.; Sidhu, R.; Dietzen, D. J.; Fu, R.; Wassif, C. A.; Yanjanin, N. M.; Marso, S. P.; House, J.; Vite, C.; Schaffer, J. E.; Ory, D. S. Cholesterol oxidation products are sensitive and specific blood-based biomarkers for Niemann-Pick C1 disease. *Sci. Transl. Med.* **2010**, *2*.

22. Santiago-Mujica, E.; Flunkert, S.; Rabl, R.; Neddens, J.; Loeffler, T.; Hutter-Paier, B. Hepatic and neuronal phenotype of NPC1 −/− mice. *Heliyon* **2019**, *5*.

23. Schlegel, V.; Thieme, M.; Holzmann, C.; Witt, M.; Grittner, U.; Rolfs, A.; Wree, A. Pharmacologic treatment assigned for Niemann Pick Type C1 disease partly changes behavioral traits in wild-type mice. *Int. J. Mol. Sci.* **2016**, *17*, 1866.

24. Võikar, V.; Rauvala, H.; Ikonen, E. Cognitive deficit and development of motor impairment in a mouse model of Niemann-Pick type C disease. *Behav. Brain Res.* **2002**, *132*, 1–10.

25. Williams, I. M.; Wallom, K. L.; Smith, D. A.; Al Eisa, N.; Smith, C.; Platt, F. M. Improved neuroprotection using miglustat, curcumin and ibuprofen as a triple combination therapy in Niemann-Pick disease type C1 mice. *Neurobiol. Dis.* **2014**, *67*, 9–17.
